# Immune inhibitory receptor-mediated immune response, metabolic adaptation, and clinical characterization in patients with COVID-19

**DOI:** 10.1038/s41598-023-45883-w

**Published:** 2023-11-06

**Authors:** Huaying An, Congrui Yan, Jun Ma, Jiayuan Gong, Fenghua Gao, Changwen Ning, Fei Wang, Meng Zhang, Baoyi Li, Yunqi Su, Pengyu Liu, Hanqi Wei, Xingwei Jiang, Qun Yu

**Affiliations:** 1 Institute of Health Service and Transfusion Medicine, Academy of Military Medical Sciences, Beijing, China; 2 Department of Cardiology, Chinese People’s Liberation Army Lanzhou General Hospital Anning Branch, Lanzhou, China

**Keywords:** Viral infection, Predictive markers, Prognostic markers, Transcriptomics

## Abstract

Immune inhibitory receptors (IRs) play a critical role in the regulation of immune responses to various respiratory viral infections. However, in coronavirus disease 2019 (COVID-19), the roles of these IRs in immune modulation, metabolic reprogramming, and clinical characterization remain to be determined. Through consensus clustering analysis of IR transcription in the peripheral blood of patients with COVID-19, we identified two distinct IR patterns in patients with COVID-19, which were named IR_cluster1 and IR_cluster2. Compared to IR_cluster1 patients, IR_cluster2 patients with lower expressions of immune inhibitory receptors presented with a suppressed immune response, lower nutrient metabolism, and worse clinical manifestations or prognosis. Considering the critical influence of the integrated regulation of multiple IRs on disease severity, we established a scoring system named IRscore, which was based on principal component analysis, to evaluate the combined effect of multiple IRs on the disease status of individual patients with COVID-19. Similar to IR_cluster2 patients, patients with high IRscores had longer hospital-free days at day 45, required ICU admission and mechanical ventilatory support, and presented higher Charlson comorbidity index and SOFA scores. A high IRscore was also linked to acute infection phase and absence of drug intervention. Our investigation comprehensively elucidates the potential role of IR patterns in regulating the immune response, modulating metabolic processes, and shaping clinical manifestations of COVID-19. All of this evidence suggests the essential role of prognostic stratification and biomarker screening based on IR patterns in the clinical management and drug development of future emerging infectious diseases such as COVID-19.

## Introduction

In 2019, severe acute respiratory syndrome coronavirus 2 (SARS-CoV-2) caused coronavirus disease 2019 (COVID-19) and rapidly escalated to the twenty-first century’s deadliest pandemic. At present, the number of infected cases has exceeded 755 million, with more than 6.8 million deaths as a result of infection^1^. Clinical symptoms of SARS-CoV-2 infection range from asymptomatic to life-threatening. Approximately 5% of patients develop severe disease that requires intensive care with mechanical ventilation^2,3^. Epidemiological studies attempt to identify risk factors for severe disease. Immune imbalances, such as dysregulated innate immune responses, early immunosuppression, lymphopenia, and cytokine storms, are considered one of the causes of life-threatening COVID-19^4^.

Homeostasis is key for biological systems to maintain health. Upon infection, the SARS-CoV-2 virus disturbs the homeostasis of the physiological system, following which, the immune system needs to rapidly recognize the disturbance and restore homeostasis as quickly as possible before the organism develops immunopathology. In patients with COVID-19, delayed early antiviral immune response leads to uncontrolled virus replication. Persistent viral exposure and antigen presentation may result in excessive production of cytokines and complement, remarkably decreased immune cell numbers, and exhaustion of host cellular immune responses^5^. Such circumstances are harmful for patients with COVID-19 because of the increasing risk of developing immunopathology.

Therefore, our immune system has evolved an extensive immune regulatory network, which is tightly regulated by both activating and inhibitory immune receptors (IRs). The physiological role of IRs is primarily to maintain immune homeostasis and tolerance^6,7^. More importantly, IRs can fine-tune immune responses in a highly specific manner to prevent the occurrence of immunopathology^8^. An increasing number of studies demonstrate that IRs are indispensable modulators of the immune system during the development of COVID-19^9–12^. Many studies have discovered that programmed cell death-1 (PD-1)^10^, cytotoxic T lymphocyte-associated antigen (CTLA-4), T cell immunoreceptor with Ig and ITIM domains (TIGIT)^10,11^, and killer cell immunoglobulin-like receptor 2DL1 (KIR2DL1)^9^ are upregulated during the development of severe COVID-19 disease. Another study suggests that the expression of T-cell immunoglobulin mucin-3 (TIM-3, also named HAVCR2) and lymphocyte-activation gene-3 (LAG3) in patients with more severe COVID-19 is much higher than that in patients with mild disease. However, both inhibitory receptors rapidly decline to normal levels during convalescence^13^. In addition, the upregulation of TIM-3 can induce the depletion of NKT cells, which is related to disease severity and prognosis of patients with COVID-19. Compared to healthy individuals, in patients with COVID-19, TIM-3^+^ NKT cells present high levels of co-inhibitory receptors such as PD-1, CTLA4, and LAG3^14^. To clarify the expression profile of more IRs during SARS-CoV-2 infection, Narjes et al. performed bioinformatics analyses to determine the expression levels of specific IRs in different types of samples, identified eight upregulated IRs in both nasopharyngeal swabs and autopsies and determined their correlations with viral loads^15^. These studies suggest that the elevated expression of IRs in patients with severe COVID-19 contribute to the exhaustion of effector immune cells and the impairment of their specific immune activity. However, some studies have reported that CD8^+^ T cells with high expression of PD-1 and CTLA-4 are not exhausted but functional^12,16^. Some researchers have also proposed that these IRs are upregulated in effector T cells to protect vital organs from the excessive inflammatory environment triggered by SARS-CoV-2 infection^17^, which suggests an immune activation-induced negative feedback mechanism of IRs. Therefore, the mechanism by which IRs regulate the immune system to control infection in COVID-19 remains controversial.

Although the above findings provide clues to the correlation between dysregulated IRs and the severity of COVID-19, most of these studies focused on only one or two IRs and cell types. However, severe disease is characterized by an immune disorder tuned by the integrated effect of multiple IRs. Therefore, there is an urgent need to explore the integrated impact of multiple IRs on individual patients with severe COVID-19. Establishing an IRscore based on principal component analysis of IR-related genes is an effective method to comprehensively evaluate the association between IR crosstalk and the severity of COVID-19. Blood samples are ideal for easy acquisition and biomarker screening, which can provide the genetic landscape of disease states and the opportunity to systematically track disease progression^18^. It is essential to conduct data analysis on blood sample transcriptomes to evaluate disease states and to screen effective diagnostic and therapeutic biomarkers.

In this study, we used transcriptome sequencing data from COVID-19 peripheral blood mononuclear cell (PBMC) samples to comprehensively evaluate the correlation between IR expression patterns and patient prognosis and immune and metabolic characteristics. We then established a scoring system, IRscore, to quantify the IR expression pattern in individual patients with COVID-19. Finally, we verified the reliability and stability of the IRscore in predicting the disease state, clinical characteristics, and immunotherapeutic efficacy of COVID-19. A comprehensive understanding of the role of the IR regulatory network in modulating the immune response and shaping clinical manifestations could enhance our understanding of COVID-19 progression and provide avenues for discovering effective therapeutic targets.

## Methods

### Sources of the COVID-19 datasets

The datasets analyzed during the current study are available in the GEO repository were GSE157103 (https://www.ncbi.nlm.nih.gov/geo/query/acc.cgi?acc=GSE157103), GSE198256 (https://www.ncbi.nlm.nih.gov/geo/query/acc.cgi?acc=GSE198256), GSE152418 (https://www.ncbi.nlm.nih.gov/geo/query/acc.cgi?acc=GSE152418), and GSE163317 (https://www.ncbi.nlm.nih.gov/geo/query/acc.cgi?acc=GSE163317). The basic information of these datasets is summarized in Table S1.

The RNA-seq data and clinical information of 126 PBMC samples were obtained from the GSE157103 dataset^19^. Among the 126 patients, 100 were patients with COVID-19 and the others were patients without COVID-19. The normalized transcripts per million was downloaded to study the potential clinical value of immune inhibitory receptors. This dataset included clinical information such as age, sex, hospital-free days at day 45 (HFD-45), ICU, mechanical ventilator support, sequential organ failure assessment score, Charlson comorbidity index score, and clinical laboratory parameters. The clinical information is summarized in Table S2.

To assess the stability of the IRscore to predict the disease state of COVID-19, we included the GSE198256 and GSE152418 datasets. The GSE198256 dataset comprised 11 healthy controls, 7 patients with acute infection, and 16 convalescents. Monocytes isolated from the PBMCs of these patients were sequenced using NovaSeq 6000^20^. Normalized counts were downloaded. RNA-seq analysis of the PBMCs from 17 patients with COVID-19 and 17 healthy controls was included in the GSE152418 dataset. Among the patients with COVID-19, 1 was a convalescent patient, 4 were patients with moderate disease, and 12 were patients with severe disease^21^. The counts were downloaded.

To determine the relationship between IRscore and therapeutic effects, we collected the GSE163317 dataset with treatment information, which included RNA-seq data from whole blood samples of 4 patients with severe COVID-19 before and 7 days after anakinra (IL-1 receptor blocker) administration^22^.

### Principal component analysis

Principal component analysis (PCA) was performed on the transcriptome gene expression data using the prcomp function with default parameters^23^. The PCA plots were grouped by distinct IR patterns.

### Unsupervised clustering of 42 immune inhibitory receptors

We used the createDataPartition function in the “caret” R package to split 100 patients with COVID-19 in the GSE157103 dataset into a training cohort with 75 patients and a test cohort with 25 patients^24^. To balance the sample distributions within the splits, we conducted random sampling within the levels of HFD-45 outcomes. The training cohort with 75 patients with COVID-19 was used for model training, and the test cohort with 25 samples was used to evaluate model performance.

The human genome contains more than 300 potential inhibitory receptor genes, although only 60 of these have been functionally demonstrated over the past few decades^7,8^. We selected 42 immune inhibitory receptors (IRs) that Narjes^15^ and Rumpret^8^ incorporated in their research to identify distinct IR patterns in COVID-19. We applied unsupervised clustering analysis based on the expression of 42 IRs to classify patients with COVID-19 in the training cohort for further analysis. The unsupervised clustering analysis and the stability of grouping were determined using the consensus clustering algorithm performed using the “ConsensusClusterPlus” R package^25^. To guarantee the stability of classification, we assigned the number of resamplings as 1000.

### Gene set variation analysis and functional annotation

To identify the discrepancy in biological processes between IR patterns, we executed GSVA enrichment analysis using the “GSVA” R package. GSVA is commonly applied to estimate variations in signaling pathways and biological processes over a population using a non-parametric and unsupervised method^26^. The gene sets “c2.cp.kegg.v7.1.symbols” and “c5.go.v7.5.1.symbols” were downloaded from the MSigDB database for GSVA analysis (http://www.gsea-msigdb.org/gsea/downloads.jsp). The *P* value was adjusted using the Benjamini and Hochberg method^27^. The pathway with an adjusted *P* value of 0.01 was considered statistically significant. The “clusterProfiler” R package was used to annotate the function of IR-related differentially expressed genes (DEGs), with 0.01 as the FDR cutoff value^28^.

### Estimation of immune cell fractions

To quantify the proportions of innate and adaptive immune cells in COVID-19 samples, we used the CIBERSORT algorithm to estimate the abundances of member cell types in a mixed cell population (https://cibersort.stanford.edu). CIBERSORT is a deconvolution algorithm that uses support vector regression and a set of reference gene expression values representing several human immune cell phenotypes to infer member cell proportions from the gene expression data of blood samples with mixed cell types. The LM22 signature gene matrix and RNA-seq data of the samples were used as inputs^29^.

### Metabolic expression subtype classification

Metabolic expression subtype classification was performed according to the method developed by Peng et al.^30^. The gene sets of the seven metabolic pathways were based on the latest Reactome annotations^31^. The seven metabolic pathways comprised lipid metabolism (766 genes), amino acid metabolism (348 genes), carbohydrate metabolism (286 genes), vitamin cofactor metabolism (168 genes), TCA cycle (148 genes), energy integration (110 genes), and nucleotide metabolism (90 genes). We first normalized gene expression across samples by Z score to obtain a rank value for each gene (~ 19,000 coding genes) within each sample. We then conducted gene set enrichment analysis (GSEA) based on the gene set of a specific metabolic pathway. We classified samples into three subtypes according to the resulting rank values. The classification criteria were as follows: (1) samples with high Z scores for a specific metabolic pathway were defined as “upregulated subtype” (FDR < 0.05); (2) samples with low Z scores for a specific metabolic pathway were defined as “downregulated subtype” (FDR < 0.05); and (3) samples showing no significant enrichment pattern were defined as “neutral subtype.” Given the metabolic expression subtypes, we evaluated the expression alteration of protein-coding genes using the Kruskal–Wallis test and then used the Kolmogorov–Smirnov test to determine whether the *P* values of the metabolic pathway genes were lower than those of other genes (FDR < 0.05). Metabolic pathways with FDR > 0.05 were defined as non-significant metabolic pathways and excluded from further analyses. Each sample was then labeled with a metabolic pathway subtype using significant metabolic pathways. Differences in the proportions of each metabolic expression subtype between the two IR clusters were statistically analyzed using the chi-square test.

### Generation of the IRscore

To quantify the IR patterns of individual patients with COVID-19, we established a scoring system termed IRscore. The model constructed in the training cohort was validated in the test cohort. The procedures for constructing this model were as follows.

#### Acquisition of significant DEGs

First, the analysis of RNA-Seq counts of the training cohort was conducted using the “DESeq” package to detect differentially expressed genes between the two IR patterns^32^. DEGs identified from distinct IR patterns were extracted to classify patients. The unsupervised consensus clustering algorithm was used to determine the number of gene clusters and the stability of clustering. Second, we divided patients with COVID-19 according to the days of no hospitalization in 45 days; those with hospitalization days more than 26 days were named as HFD-45 > 26, and the rest were named as HFD-45 ≤ 26. DEGs between the two groups were acquired using the “DESeq” package. Third, we identified DEGs between patients with and without COVID-19. The DEGs in each of the above three comparison groups were determined by significance criteria, which retained DEGs with adjusted *P* values < 0.05 and log2 fold change > 1 for the following analyses. Overlap analyses were conducted to extract overlapping DEGs from the above three differential expression analysis results (Fig. S4M). Finally, the random forest algorithm was adopted to perform dimension reduction to reduce noise and redundant genes. The random forest classification algorithm was implemented using the R package Boruta^33^.

#### Construction of the IRscore

The expression of each gene was first transformed into a Z score, before conducting PCA to construct an IR-relevant gene signature. Both principal components 1 and 2, which reflect the main characteristics of IR and prognosis-related DEGs, were extracted as signature scores. We then applied a method similar to the gene expression grade index (GGI) to define the IRscore of each patient^34^:

$${\text{IRscore}} = \Sigma ({\text{PC}}1_{{\text{i}}} + {\text{PC}}2_{{\text{i}}} )$$

where i is the IR phenotype-related gene.

### Lipopolysaccharide-induced inflammatory model

An LPS-induced inflammatory model was established as described previously^35^. Eight-week-old male C57BL/6J mice weighing 22–24 g were purchased from Charles River Laboratories. All mice were maintained in pathogen-free cages at 24 °C under a 12-h light–dark cycle. We randomly divided the mice into two groups: the control group (n = 6) and the LPS group (6 mg/kg, n = 14). LPS (*Escherichia* *coli* O55:B5, Sigma, St. Louis, MO, USA) was dissolved in sterile saline and administered intraperitoneally. The control group received sterile saline. Mice were weighed 24 h after LPS administration. The mice were euthanized by cervical dislocation, and the lung and spleen were collected. The whole lungs of the mice were weighed, and the ratio of lung weight to body weight was used to determine the success of the LPS-induced inflammation model. Lung tissue samples were embedded in paraffin and subjected to hematoxylin and eosin (H&E) staining. No animal anesthesia was used in any experiment. All mice experiments were conducted according to standard protocols approved by the Animal Ethics Committee of the Institute of Health Service and Transfusion Medicine (application number: IACUC-DWZX-2023-P657). This study is reported in accordance with the ARRIVE guidelines (https://arriveguidelines.org).

### Spleen lymphocyte isolation

The spleens were processed by mechanical disruption using a 40-μM mesh filter. Saline with suspended spleen cells was transferred to a 3 mL lymphocyte separation medium (7211011, Dakewe, China) and centrifuged at 800*g* at room temperature for 30 min, with a slower lifting and descending speed. After centrifugation, we collected the lymphocyte layer and washed the cells with 10 mL of saline. Finally, the cells were collected by centrifugation at 250*g* for 10 min at room temperature.

### RNA extraction and real-time polymerase chain reaction

Total RNA from the isolated spleen lymphocytes was extracted using TRIzol reagent (15596026, Invitrogen, Thermo Fisher Scientific, USA). Total RNA (2 mg) was reverse transcribed using All-in-one RT MasterMix (G592, abm, Canada) according to the manufacturer’s instructions. The real-time polymerase chain reaction (real-time PCR) was performed using UltraSYBR Mixture (CW0957S, CWBIO, China) on LightCycler96 (Roche, Switzerland). The real-time PCR primers are listed in Supplementary Table S3.

### H&E staining

The lung tissue in the control and LPS groups at 24 h post-LPS administration were fixed in 4% formaldehyde. H&E staining was performed according to the methods reported in a previous study^36^. The pathological photos were taken using a microscope (Olympus, Japan).

### Statistical analysis

Correlation coefficients between CIBERSORT member cells and expression of IR genes were calculated by Spearman’s correlation analysis. Mann–Whitney and Student’s *t* tests were applied to conduct difference comparisons between the two groups. One-way analysis of variance (ANOVA) with Dunnett’s multiple comparison test was used to compare differences among three or more groups of data subject to normal distribution. A heatmap of antiviral innate immune responses was constructed on the DEGs between two IR patterns belonging to the GO:0140374 annotations (http://geneontology.org, Table S4). A heatmap of the major histocompatibility complex was constructed on the DEGs between two IR patterns according to a publication by Zhang et al.^37^. In light of the correlation between IRscore and mechanical ventilator support state, the optimal cut point of the dataset subgroup was determined by the surv_cutpoint function in the “survminer” R package^38^. This is an outcome-oriented method that provides a value of a cutpoint that corresponds to the most significant relation with the outcome. Therefore, we implemented this algorithm to dichotomize patients into low- and high-IRscore groups based on the maximally selected rank statistics. We adopted a multivariate Cox regression model to determine the independent prognostic factors for evaluating the outcomes of patients with COVID-19^39^. The “forestplot” R package was employed to visualize the results of multivariate Cox regression analysis for the IRscore in the training cohort^40^. The performance (sensitivity and specificity) of the IRscore in predicting the mechanical ventilator support state of patients with COVID-19 was evaluated by receiver operating characteristic (ROC) curves, which were plotted through the area under the curve (AUC) scores calculated by “survivalROC”^41^. All statistical analyses were conducted using R (https://www.r-project.org, R version 4.0.4) or SPSS software (version 26.0.0.0). Two-sided *P* values < 0.05 were considered statistically significant.

## Results

### Expression level of 42 immune inhibitory receptors was associated with the characteristics of immune cell abundance and disease state in patients with COVID-19

**Figure 1:**
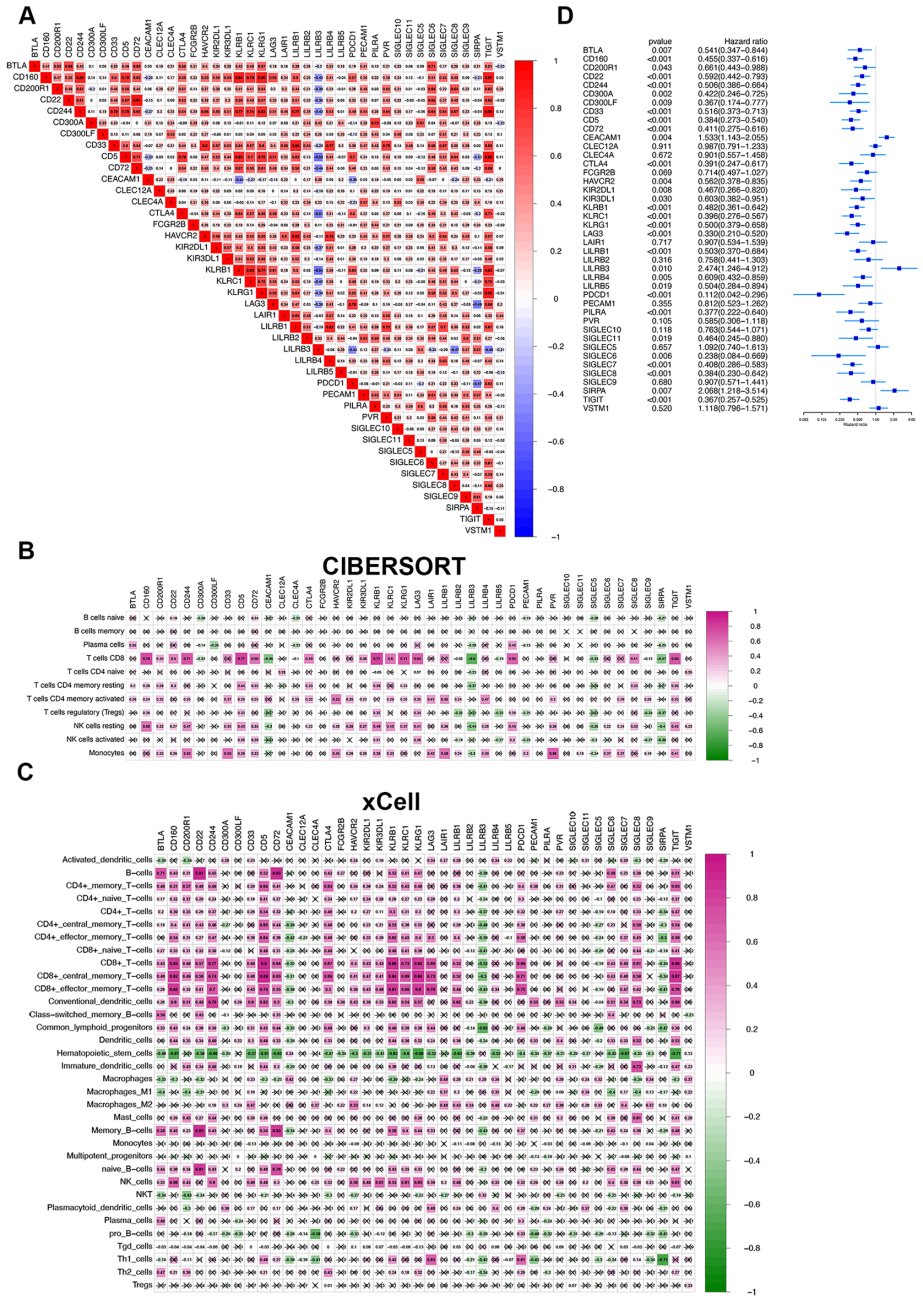
Prognostic characterizations of 42 IRs and their associations with immune cell abundances in peripheral blood mononuclear cells from patients with COVID-19. (**A**) Correlation analyses for 42 IRs in 100 patients with COVID-19 using the Spearman method. Negative correlation is marked in blue and positive correlation is marked in red. (**B**) Correlation between the components of 11 immune cells calculated by CIBERSORT and 42 IRs in 100 patients with COVID-19. Negative correlation is marked in green and positive correlation is marked in violet red. (**C**) Correlation between the components of 34 immune cells calculated by xCell and 42 IRs in 100 patients with COVID-19. Negative correlation is marked in green and positive correlation is marked in violet red. (**D**) Prognostic analyses of 42 IRs in 100 patients with COVID-19 using a univariate Cox regression model. A hazard ratio > 1 represented risk factors for the requirement of ventilator support, while a hazard ratio < 1 represented protective factors for requiring ventilator support. *IR* immune inhibitory receptors.

We reviewed the available literature on functionally characterized inhibitor receptor–ligand pairs and selected 42 immune inhibitory receptors (IRs) to identify distinct IR patterns. We first conducted correlation analysis to identify the interaction patterns of these IRs in the GSE157103 dataset. As shown in Fig. 1A, most IRs displayed synergistic expression, whereas LILRB3 exhibited antagonistic action with most IRs. Referring to the distribution of inhibitory receptors on immune cells summarized by Narjes et al.^15^, we surprisingly found that IRs with strong correlations (coefficient > 0.8) were primarily expressed on the same type of immune cells (Table 1). Specifically, TIGIT, CD244, and CD160 with high correlation coefficients were all distributed on NK and T cells (TIGIT and CD244 correlation coefficient: 0.883, TIGIT and CD160 correlation coefficient: 0.872, CD244 and CD160 correlation coefficient: 0.844, Fig. 1A). We also estimated the abundances of member cell types in the blood lymphocytes of patients with COVID-19 using the CIBERSORT and xCell method and determined the association between IRs and the abundance of immune cells. CD8^+^ T cells were positively correlated with the expression levels of CD160, CD200R1, CD22, CD244, CD5, CD72, CTLA4, KLRB1, KLRC1, KLRG1, LAG3, PDCD1, SIGLEC8, and TIGIT, whereas their abundances were negatively correlated with LILRB3 (Fig. 1B,C). We then used a univariate Cox regression model to calculate the hazard ratio of each IR in the PBMCs of patients with COVID-19. The forest plot displayed the 42 IR prognostic values for ventilator-free intervals in patients with COVID-19 (Fig. 1D), indicating a possible association between IR expression levels and disease state. The above results indicated that crosstalk among 42 IRs might play considerable roles in the formation of different IR patterns, the characterization of PBMC immune cell abundance or composition, and the prediction of disease states in individual patients.

**Table 1:** List of immune inhibitory receptors with correlation coefficients ≥ 0.800 and their predominant colocalization on immune cells.

Gene1	Gene2	Correlation coefficient	Cell colocalization^1^
HAVCR2	CD33	0.800	Monocytes, macrophages
LILRB1	HAVCR2	0.816	Monocytes, macrophages, and NK cells
LILRB4	LILRB1	0.824	Monocytes, macrophages, neutrophils, and DCs
TIGIT	KLRB1	0.827	NK cells
KLRC1	KLRB1	0.827	NK cells
KLRB1	CD5	0.830	Not located on the same cells
KLRG1	CD244	0.831	NK cells
KLRB1	CD160	0.843	NK cells
CD244	CD160	0.844	NK and T cells
TIGIT	KLRG1	0.850	NK cells
KLRG1	CD160	0.856	NK cells
LILRB1	CD33	0.860	Monocytes, macrophages
TIGIT	CD160	0.872	NK and T cells
TIGIT	CD5	0.880	T cells
TIGIT	CD244	0.883	NK and T cells
CD72	CD22	0.909	B cells

^1^The cell colocalization was taken from a publication by Narjes et al.^15^.

### IR patterns mediated by 42 immune inhibitory receptors and clinical characteristics of each pattern

**Figure 2:**
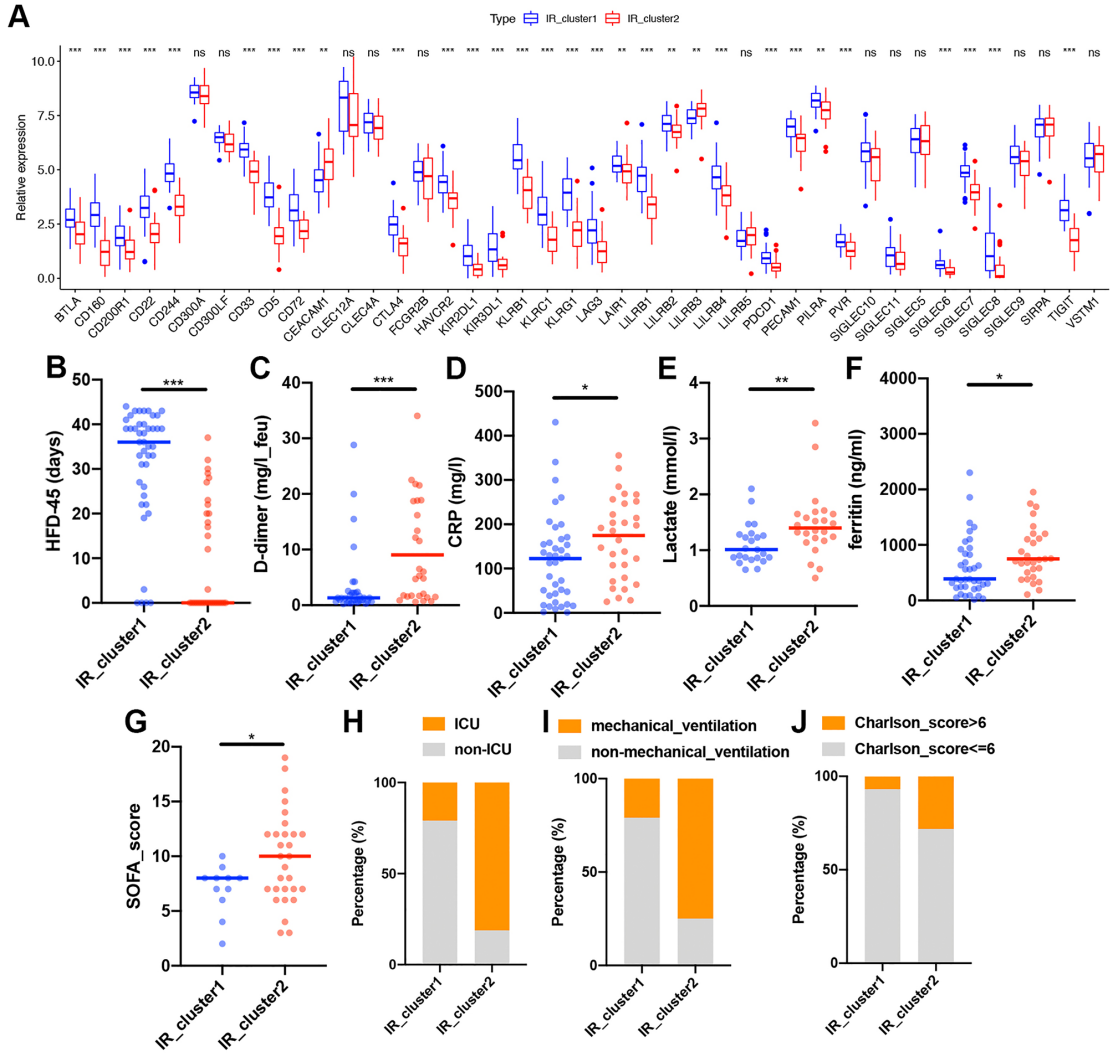
IR patterns mediated by 42 inhibitory receptors and clinical characteristics of each pattern. (**A**) Expression levels of 42 IR genes between two IR clusters in the training cohort (Mann–Whitney *U* test). The upper and lower ends of the boxes represent the interquartile range of values. The lines in the boxes represent median values, and the dots show outliers. (**B**) Differences in the hospital-free days at day 45 (HFD-45) between the two IR clusters in the training cohort (Mann–Whitney *U* test). (**C**–**F**) Differences in the concentrations of D-dimer (**C**), CRP (**D**), lactate (**E**), and ferritin (**F**) between the two IR clusters in the training cohort (Mann–Whitney *U* test). (**G**) Differences in the SOFA scores between the two IR clusters in the training cohort (unpaired *t*-test). (**H**–**J**) Differences in the proportions of ICU (**H**), mechanical ventilation (**I**), and Charlson score > 6 (**J**) between the two IR clusters in the training cohort (chi-square test). Asterisks represent the statistical *P* value (*P < 0.05; **P < 0.01; ***P < 0.001; *ns* no significance).

Regarding the crosstalk of 42 IRs in the PBMCs of patients with COVID-19, we performed consensus clustering to classify patients into appropriate subgroups with different IR patterns based on the expression levels of 42 IRs. Two distinct patterns were identified, with 43 cases in cluster 1 and 32 cases in cluster 2; these patterns were termed IR_cluster1 and IR_cluster2, respectively. The cluster heatmaps displayed the stability of the two clusters (Figs. S1A–C). Principal component analysis (PCA) revealed a clear distinction between the two patterns (Fig. S1D). A unique IR transcriptional profile was observed between the two IR patterns. IR_cluster2 displayed significantly high expression of CEACAM1 and LILRB3, whereas IR_cluster1 was characterized by markedly elevated expression of most other IRs (Fig. 2A).

We then investigated the differences in disease state between the two IR patterns. As shown in Fig. 2B, the average hospital-free days at day 45 (HFD-45) of patients in IR_cluster2 were significantly shorter than that of patients in IR_cluster1, suggesting a worse prognosis for those in IR_cluster2. Consistent with the poor disease state of IR_cluster2, more patients in IR_cluster2 required intensive care unit (ICU) admission (20.9% vs. 81.3%) and mechanical ventilator support (20.9 vs. 75.0%, Fig. 2H,I). In addition, 28.1% of patients in IR_cluster2 had a Charlson Comorbidity Index (CCI) > 6 compared to only 7.0% in IR_cluster1 (Fig. 2J), indicating the worse overall burden of comorbidities in IR_cluster2. Moreover, the higher levels of biomarkers for the diagnosis and monitoring of COVID-19 (e.g., D-dimer, C-reactive protein [CRP], lactate [LDH], and ferritin) suggested that IR_cluster2 patients showed more severe disease (Fig. 2C-F). The high Sequential Organ Failure Assessment (SOFA) scores for IR_cluster2 patients also indicated more severe organ dysfunction or failure (Fig. 2G). We also observed that IR_cluster2 was predominated by male (female vs. male, 55.8% vs. 65.6%) and elderly patients (Fig. S1E,F). All of the abovementioned evidence suggested that the essential role of IR expression patterns in predicting the prognosis and severity of patients with COVID-19 revealed a prominent survival disadvantage in IR_cluster2.

### Immunological features of distinct IR patterns in patients with COVID-19

**Figure 3:**
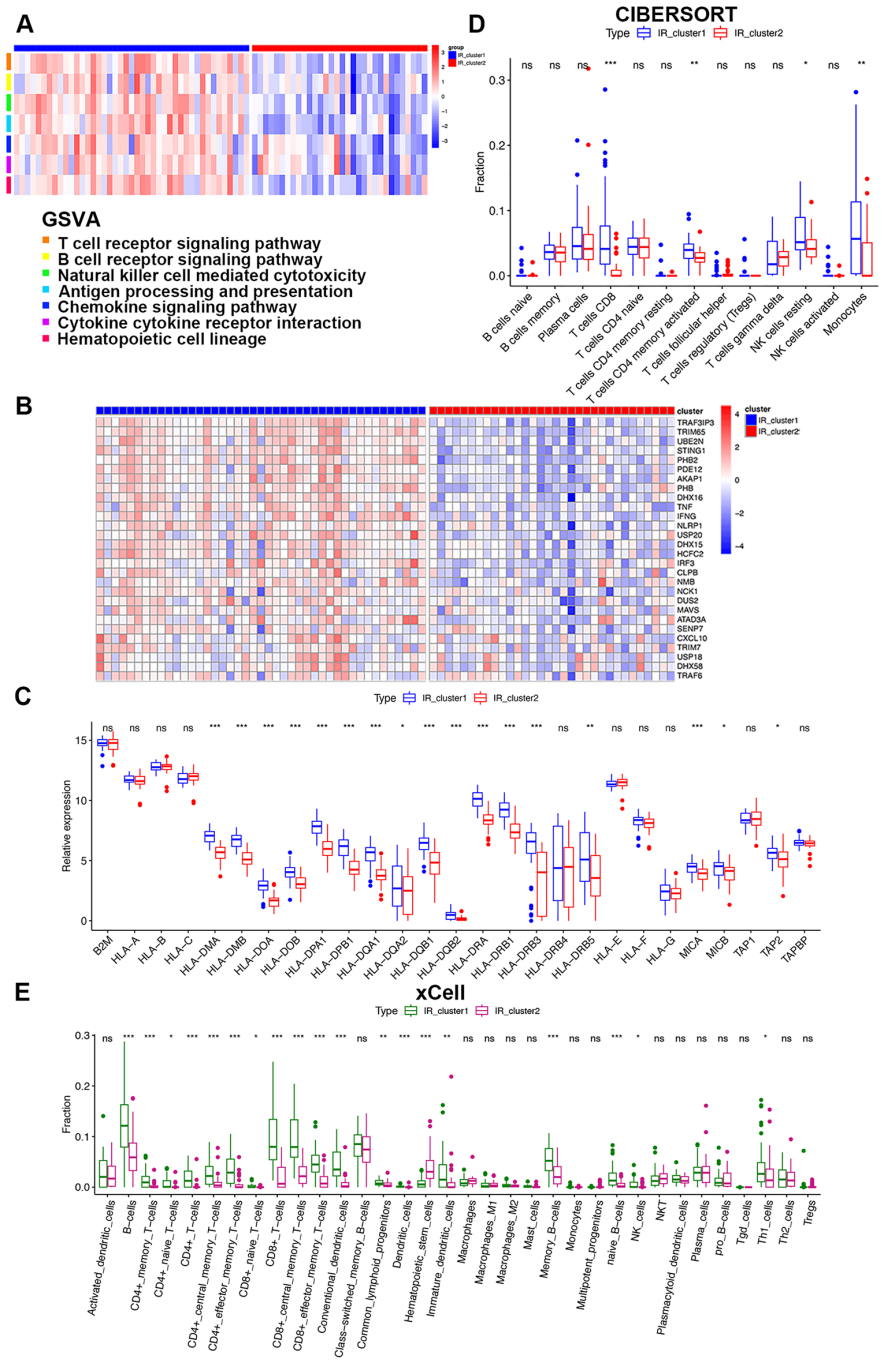
Immune response characteristics between two distinct IR patterns. (**A**) GSVA enrichment analysis showing the activation of immune pathways in distinct IR patterns in the training cohort. The heatmap was used to visualize the enrichment differences of these processes between the two IR patterns, where red represents activated pathways and blue represents inhibited pathways. (**B**) Heatmap of antiviral innate immune response pathway DEGs between the two IR clusters in the training cohort. Red represents high expression levels, and blue represents low expression levels. The IR cluster was used for patient annotations. Each column represents one patient and each row represents one gene associated with the innate immune signaling pathway. (**C**) Expression levels of MHC-related genes between the two IR clusters in the training cohort (Mann–Whitney *U* test). (**D**) The abundance of immune cells calculated by CIBERSORT between distinct IR patterns in the training cohort (Mann–Whitney *U* test). (**E**) The abundance of immune cells calculated by xCell between distinct IR patterns in the training cohort (Mann–Whitney *U* test). Asterisks represent the statistical *P* value (*P < 0.05; **P < 0.01; ***P < 0.001; *ns* no significance).

We next performed GSVA enrichment analysis to explore the biological functions of these distinct IR patterns. As expected, the significant discrepant enrichment primarily focused on immune signaling pathways. Specifically, IR_cluster1 presented significant enrichment in pathways associated with immune activation, such as the T cell receptor signaling pathway, B cell receptor signaling pathway, natural killer cell-mediated cytotoxicity, antigen processing and presentation, and chemokine signaling pathway (Fig. 3A). The immune system is broadly divided into two parts: the innate and adaptive immune systems. During viral infection, the innate immune system rapidly recognizes the infection, triggers the “alarm bells” of the type I interferon (IFN) signaling pathway, and induces the production of downstream antiviral proteins and other molecules^42^. We analyzed the expression of antiviral innate immune response signaling pathway genes between different IR patterns and observed a significantly diminished antiviral innate immune response in IR_cluster2 patients (Fig. 3B). We also discovered remarkable decreases in major histocompatibility complex (MHC)-related genes in IR_cluster2 patients (Fig. 3C). Huang et al. documented that most patients with severe COVID-19 displayed enhanced levels of proinflammatory cytokines^43^. Therefore, we evaluated the expression levels of these cytokines between the two IR patterns. As shown in Fig. S1G, S100A12, a marker of inflammation in severe sepsis, was dramatically augmented in IR_cluster2 patients, further indicating that IR_cluster2 patients may develop pulmonary injury and endure severe disease burden.

Although the innate and adaptive immune systems are linked in multitudinous important ways, they each comprise distinct cell types with specific functions. B cells, CD4^+^ T cells, and CD8^+^ T cells are the three major adaptive immune cell types that control and clear viral infections. Both CIBERSORT and xCell immune cell component analyses showed that patients in IR_cluster2 had lower levels of memory CD4^+^ T cells and CD8^+^ T cells (Fig. 3D,E), suggesting the prominently suppressed T cell-dependent adaptive immune responses in IR_cluster2. All of the above-mentioned results indicated lower levels of innate and adaptive immune responses in IR_cluster2 patients, who may be more vulnerable to infection and disease progression. Therefore, the differences in infection control for IR pattern stratification patients might be attributed to IR pattern-mediated discrepancies in the landscape of immune response. Meanwhile, patients with COVID-19 with lower expression of most IRs presented with the absence of CD8^+^ T cells and worse prognosis, indicating that IR might shape the clinical characteristics of COVID-19 through an immune activation-induced negative feedback mechanism.

### Immunometabolic adaptation characteristics in distinct IR patterns of patients with COVID-19

**Figure 4:**
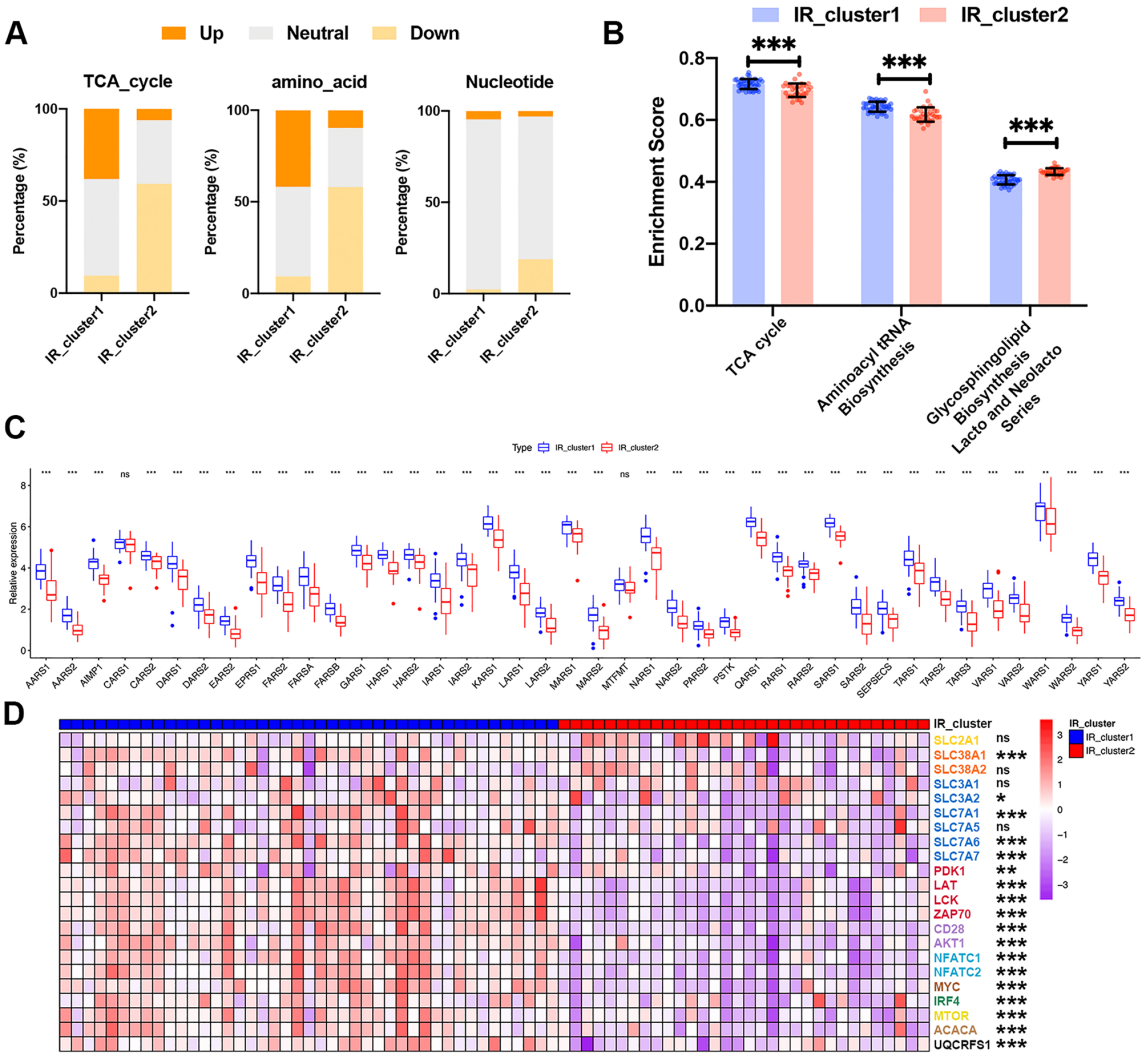
Metabolic adaptation features between two distinct IR patterns. (**A**) Differences in the proportions of upregulated, neutral, and downregulated TCA cycle metabolism, amino acid metabolism, and nucleotide metabolism between the two IR clusters in the training cohort (chi-square test). Orange represents upregulated metabolism, gray represents unchanged metabolism, and yellow represents downregulated metabolism. (**B**) Enrichment of metabolic pathways in distinct IR patterns was obtained through GSVA enrichment analysis. The bar chart displays differences in enrichment scores of these processes between the two IR patterns. (**C**) Expression levels of ARSs between the two IR clusters in the training cohort (Mann–Whitney *U* test). (**D**) Heatmap of genes linking effector T cell activation with metabolic pathways between the two IR patterns in the training cohort. Red represents high expression levels, and purple represents low expression levels. The IR cluster was used for patient annotations. Each column represents one patient, and each row represents one gene linking effector T cell activation with metabolic pathways. Asterisks represent the statistical *P* value (*P < 0.05; **P < 0.01; ***P < 0.001; *ns* no significance).

In addition to immune signaling pathways, we noticed specific metabolic pathway enrichment discrepancies between the two IR patterns. To further determine the metabolic differences between distinct IR patterns, we used a computational method to classify individual patients into “directional” metabolic pathway subtypes^30^. Among the seven metabolic pathways (lipid, amino acid, carbohydrate, vitamin cofactor, TCA cycle, nucleotide, and energy pathways), the *P* value of energy metabolism was not significant. Therefore, we labeled each sample with the remaining six metabolic expression subtypes. As shown in Fig. 4A, fewer patients in IR_cluster2 exhibited upregulated TCA cycle (38.1 vs. 6.3%) and amino acid metabolism (41.9 vs. 9.7%) compared to those in IR_cluster1, whereas more patients in IR_cluster2 exhibited downregulated nucleotide metabolism (2.3 vs. 18.8%). These results suggest that IR_cluster2 may be characterized by downregulated energy production, amino acid metabolism, and nucleotide metabolism compared with IR_cluster1.

Many studies have reported that precise control of cellular metabolic processes is necessary for fueling the fate decision and effector function of immune cells^44–47^. Therefore, we attempted to examine the crosstalk between immune cell abundance and cellular metabolism using Spearman’s correlation analyses. Our results showed that immune cells with significantly different abundances between distinct IR patterns were positively correlated with both tricarboxylic acid (TCA) cycle and aminoacyl tRNA biosynthesis, while negatively correlated with the Glycosphingolipid Biosynthesis Lacto and Neolacto Series (Fig. S2). Furthermore, IR_cluster2, with lower overall immune cell abundance, exhibited a lack of TCA cycle and aminoacyl tRNA biosynthesis pathway, while exhibiting enrichment in the Glycosphingolipid Biosynthesis Lacto and Neolacto Series pathway (Fig. 4B). As recent studies have suggested that aminoacyl-tRNA synthetases (ARSs) could control antiviral innate immunity and lead to viral clearance^48–51^, we analyzed the expression levels of ARSs between two IR patterns. As expected, almost all of these ARSs were downregulated in IR_cluster2 (Fig. 4C). Therefore, the decreased expression of ARSs might also represent one of the metabolic mechanisms that suppressed antiviral immune responses in IR_cluster2 patients, further verifying the potential of ARSs as therapeutic targets for infectious diseases.

We also compared the expression levels of genes that linked effector T cell activation and the metabolic processes in distinct IR patterns. These genes were summarized in a publication by Miguel et al.^44^. Notably, patients in IR_cluster2 displayed decreased expression of glycolysis modulators (PDK1, LAT, LCK, ZAP70, CD28, AKT1, NFATC1, NFATC2), nutrient uptake transporters (SLC38A1, SLC3, and SLC7 subfamily members), T cell receptor (TCR)-induced transcription factors (MYC, IRF4), nutrient-sensitive signaling complex (mTOR), regulator of fatty acid biosynthesis (ACACA), and an electron transport chain (ETC) complex III component (UQCRFS1) (Fig. 4D). Taken together, this evidence suggests a strong association between metabolic processes and adaptive immune regulation, and the specific immune metabolic characteristics in different IR patterns, indicating that the low frequency of CD8^+^ T cells in IR_cluster2 might be attributed to the suppressed nutrient metabolism mediated by immune metabolic regulators. Dysregulated metabolic pathways affect CD8^+^ T cells to adequately respond to SARS-CoV-2 infection.

### Construction of an IR signature score system for patients with COVID-19

To further determine the potential biological behavior of each IR pattern, we obtained 1036 IR phenotype-related DEGs through DESeq analysis (Table S5). GO enrichment analysis revealed that these DEGs exhibited enrichment in immune cell activation and differentiation, cytokine production and secretion, and inflammatory and immune responses (Fig. S3). We then performed unsupervised clustering analyses based on 1036 DEGs to classify patients into genomic subtypes. As expected, patients in the training cohort were stably classified into two distinct IR genomic phenotypes, named DEG_clusters1 and 2, respectively (Figs. S4A–C and S5A). Thirty-two patients were clustered in DEG_cluster1, which was related to IR_cluster1, while 43 were clustered in DEG_cluster2, which was related to IR_cluster2. We also observed a worse prognosis in DEG_cluster2 patients, similar to that in IR_cluster2. Compared to DEG_cluster1, patients in DEG_cluster2 had shorter HFD-45 (Fig. S4D). Meanwhile, DEG_cluster2 exhibited higher concentrations of D-dimer, CRP, lactate, and ferritin, and presented higher SOFA scores (Fig. S4E–I). Furthermore, patients with ICU requirements or mechanical ventilator support were mainly concentrated in DEG_cluster2 (ICU: 12.5 vs. 72.1%; mechanical ventilatory support: 12.5 vs. 67.4%; Fig. S4J,K). Furthermore, more patients in DEG_cluster2 had Charlson comorbidity index scores greater than 6 (6.3 vs. 23.3%, Fig. S4L).

**Figure 5:**
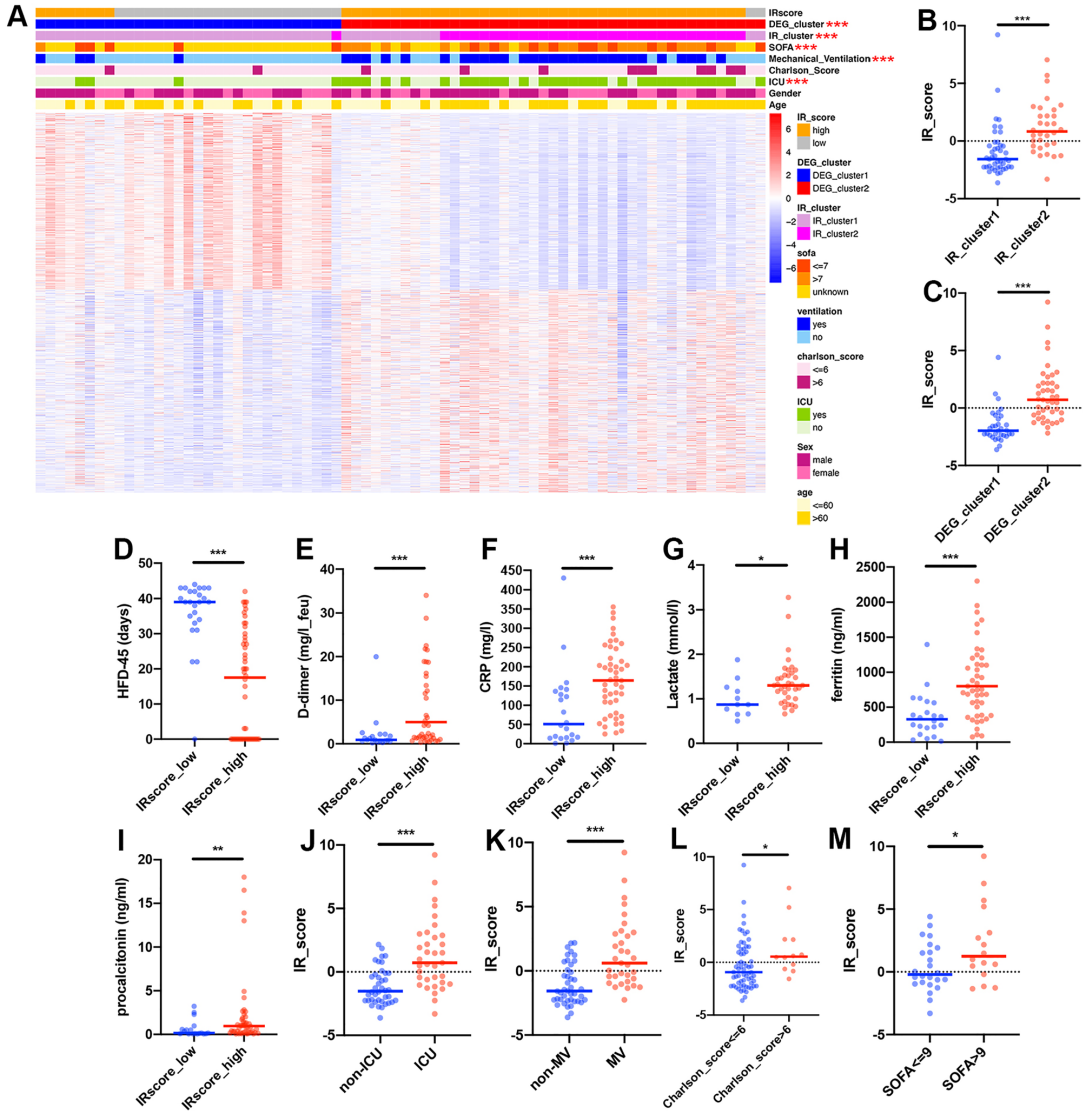
Construction of the IR signatures. (**A**) Unsupervised clustering of the IR phenotype-related genes in the training cohort to classify patients into two genomic subtypes, termed DEG_cluster 1–2, respectively. IR_cluster, IRscore, SOFA score, mechanical ventilation, Charlson score, ICU, age, and sex were used as patient annotations. Red represents high expression of phenotype-related genes, while blue represents low expression of these genes. (**B**) Differences in the IRscore between the IR patterns in the training cohort (Mann–Whitney *U* test). (**C**) IRscore between the two IR phenotype-related genomic subtypes in the training cohort (Mann–Whitney *U* test). (**D**) HFD-45 between patients with high and low IRscores in the training cohort (Mann–Whitney *U* test). (**E**–**I**) Concentrations of D-dimer (**E**), CRP (**F**), lactate (**G**), ferritin (**H**), and procalcitonin (**I**) between patients with high and low IRscores in the training cohort (Mann–Whitney *U* test). (**J**) IRscore between ICU and non-ICU patients (Mann–Whitney *U* test). (**K**) The IRscore of patients with or without mechanical ventilation (Mann–Whitney *U* test). (**L**) IRscore of patients with Charlson scores > or ≤ 6 (Mann–Whitney *U* test). (**M**) IRscore of patients with SOFA scores > or ≤ 9 (unpaired *t* test). Asterisks represent the statistical *P* value (*P < 0.05; **P < 0.01; ***P < 0.001; *ns* no significance). *MV* mechanical ventilation.

**Figure 6:**
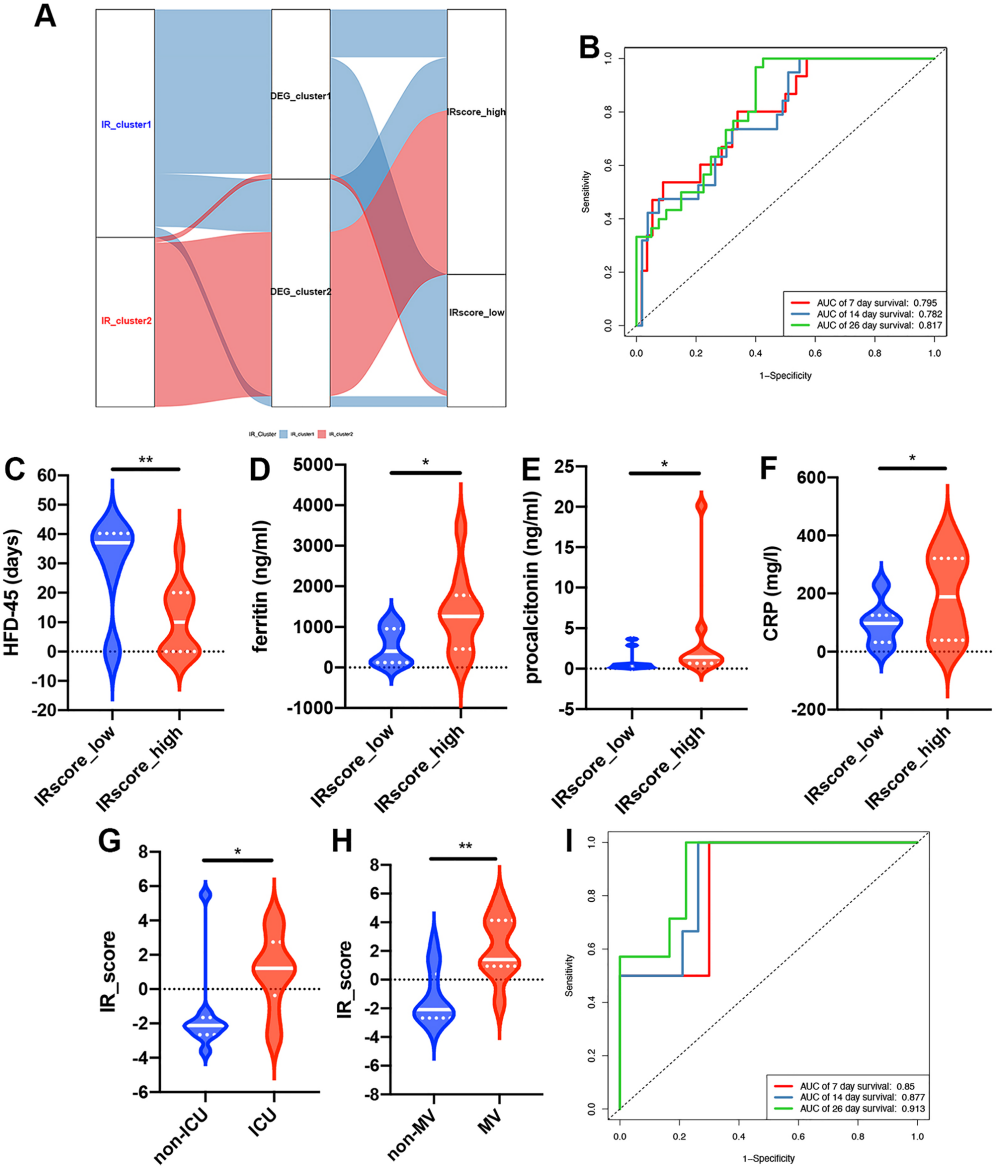
Prognostic value of IRscore for the severity of COVID-19 in test cohorts. (**A**) Alluvial diagram showing changes in IR_cluster, gene cluster, and IRscore. (**B**) The performance (sensitivity and specificity) of the IRscore on predicting the mechanical ventilator support state of patients with COVID-19 was evaluated by ROC curves. The 7-, 14-, and 26-day ROC curves of the model in the training cohort. (**C**) The HFD-45 between patients with high and low IRscores in the test cohort (Mann–Whitney *U* test). (**D**–**F**) Concentrations of ferritin (**D**, unpaired *t* test), procalcitonin (**E**, Mann–Whitney *U* test), and CRP (**F**, unpaired *t* test) between patients with high and low IRscores in the test cohort. (**G**) IRscores between ICU and non-ICU patients in the test cohort (Mann–Whitney *U* test). (**H**) IRscore of patients with or without mechanical ventilation in the test cohort (Mann–Whitney *U* test). (**I**) The performance (sensitivity and specificity) of the IRscore in predicting the mechanical ventilator support state of patients with COVID-19 was evaluated by ROC curves. The 7-, 14-, and 26-day ROC curves of the model in the testing cohort. Asterisks represent the statistical *P* value (*P < 0.05; **P < 0.01; ***P < 0.001; *ns* no significance). *MV* mechanical ventilation.

The above results again showed that IRs were essential for predicting the prognosis of patients with COVID-19. However, these findings were based on patient populations and could not directly predict the IR patterns of individual patients. To indicate the individual heterogeneity of IR patterns, we extracted overlapping differential genes and further removed redundant genes using random forest analysis. Finally, ten signature genes, including CLEC10A, CYSTM1, EPAS1, FCER1A, PTH2R, RSPO2, SCN5A, SEZ6L, TMEM52B, and TMEM92, were obtained to construct a PCA-based scoring system. We termed this model the IRscore. Using the cutoff value determined by the survminer package, we divided patients into two groups, named IRscore_high and IRscore_low. The alluvial diagram displays the attribute alterations of individual patients (Fig. 6A). The expression levels of 42 IRs between the IRscore_high and IRscore_low groups were analyzed using the Mann–Whitney *U* test. IRscore_low was characterized by markedly elevated expression of most IRs (Fig. S5B). The Mann–Whitney *U* test revealed that the IRscore of IR_cluster1 was significantly lower than that of IR_cluster2 (Fig. 5B). Meanwhile, DEG_cluster1 also presented a significantly lower IRscore compared to DEG_cluster2 (Fig. 5C), suggesting that a high IRscore is strongly linked to poor prognosis and immune inactivation-associated signatures. Therefore, we sought to determine the value of the IRscore in predicting the outcomes of patients with COVID-19. As expected, patients with a high IRscore had a shorter HFD-45 (Fig. 5D). In addition, there were considerable increases in the concentrations of D-dimer, CRP, lactate, ferritin, and procalcitonin in IRscore_high (Fig. 5E–I). In addition, patients with an ICU requirement displayed a higher IRscore compared to patients without an ICU requirement (Fig. 5J). Consistent with ICU alteration trends, patients exhibiting worse prognostic clinical features (such as mechanical ventilation support, high Charlson comorbidities index score, or high SOFA score) had a higher IRscore (Fig. 5K–M). We also used a multivariate Cox regression model to investigate whether the IRscore could serve as an independent prognostic biomarker for COVID-19. As shown in Fig. S5A, the IRscore could be considered a robust and independent prognostic biomarker for evaluating COVID-19 patient outcomes (HR: 1.20 (1.05–1.38)). Moreover, the areas under the receiver operating characteristic curve (ROC) of the training cohort on days 7, 14, and 26 were 0.795, 0.782, and 0.817, respectively, indicating that the scoring model possessed good predictive capacity (Fig. 6B). All of these results demonstrated that the IRscore can reflect IR patterns and predict clinical characteristics (such as the HFD-45, need for admission into the ICU, days spent on mechanical ventilator support, SOFA score, and Charlson comorbidity index score) of patients with COVID-19.

### Potential role of IRscore in predicting the disease state and therapeutic effect of patients with COVID-19

To identify the stability of the IRscore model, we applied the IRscore signature established in the training cohort to the testing cohort. The obtained results were consistent with those of the training set, indicating the stability of the IRscore in predicting the outcomes of patients with COVID-19 (Fig. 6C–H). IR patterns with lower expression of IRs exhibited lower levels of CD4^+^ T cells and CD8^+^ T cells in the testing cohort, further confirming the association between IR transcriptional profile and immune cell fraction changes in the training cohort (Fig. S5C,D). In addition, the association between IRscore and IR patterns was validated in the testing cohort (Fig. S5E). The areas under the ROC of the testing cohort on days 7, 14, and 26 were 0.85, 0.877, and 0.913, respectively (Fig. 6I), indicating the stability and reliability of the IRscore model.

**Figure 7:**
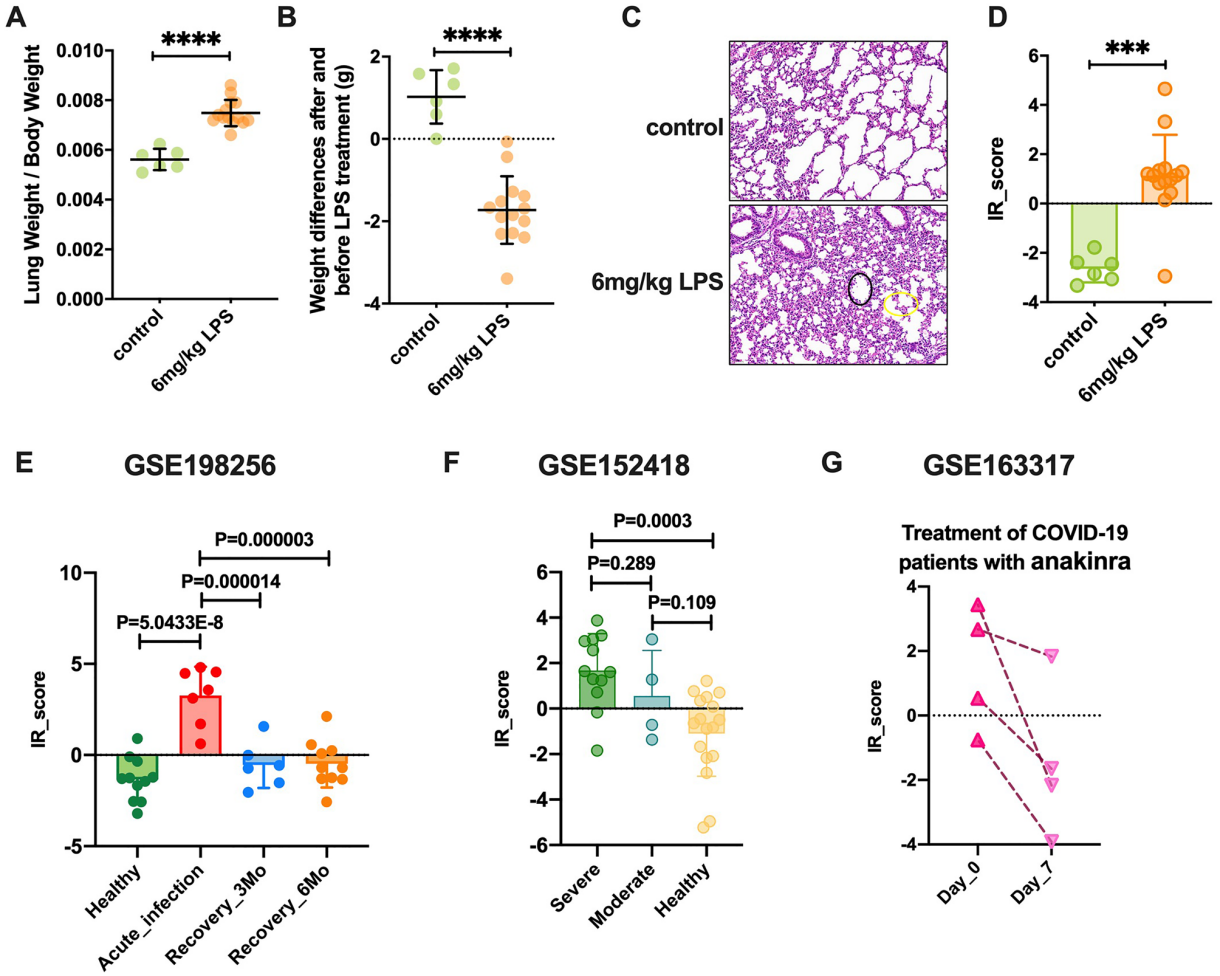
Association between IRscore and disease state and drug efficacy. (**A**) Lung/body (L/B) weight ratio in 6 mg/kg LPS (n = 14) and control (n = 6) groups. (**B**) Weight loss before and after LPS administration. (**C**) Pathological alterations in lung tissue of mice in the LPS and control groups. H&E photos were magnified by 200. (**D**) Differences in the IRscore between the control (green) and 6 mg/kg LPS (orange) groups. The Mann–Whitney *U* test was used to assess the difference. (**E**) Differences in the IRscore between acute infection and healthy and convalescence phase patients in the GSE198256 cohort. One-way analysis of variance (ANOVA) with Dunnett’s multiple comparison test was used to assess the difference. (**F**) Differences in the IRscore between severe and moderate and healthy patients with COVID-19 in the GSE152418 cohort. One-way analysis of variance (ANOVA) with Dunnett’s multiple comparison test was used to assess the difference. (**G**) Blood samples of patients with COVID-19 treated with anakinra compound were collected before and after 7 days of medication. The changes in the IRscore before and after drug treatment are shown in the line graph. Dark pink upper triangles represent the IRscore of patients with COVID-19 before anakinra treatment. Light pink lower triangles represent the IRscore of patients with COVID-19 after anakinra therapy. The IRscores of the same patient before and after treatment were connected in a straight line. Asterisks represent the statistical *P* value (*P < 0.05; **P < 0.01; ***P < 0.001; *ns* no significance).

To confirm the capability of IRscore to evaluate the clinical characteristics of patients with COVID-19, we also applied IRscore to an LPS-induced inflammation model and other independent COVID-19 cohorts with clinical information such as disease stage and state. In the LPS-induced inflammation model, higher lung-to-body weight (L/B) ratios and weight losses indicated the occurrence of pulmonary edema in, and poor physical condition of LPS-treated mice (Fig. 7A,B). The LPS group also displayed a thickened alveolar septum and marked neutrophil accumulation, which indicated the successful establishment of an inflammatory model (Fig. 7C). More importantly, we observed that the IRscore of LPS-treated mice was significantly higher than that of control mice (Fig. 7D). By applying IRscore to the GSE198256 dataset, we confirmed that IRscore could reflect COVID-19 progress (Fig. 7E). In addition, we reconfirmed the positive correlation between the IRscore and COVID-19 severity in the GSE152418 dataset (Fig. 7F). These data further verified the positive correlation between a high IRscore and multiple poor clinical characteristics of COVID-19.

Considering the close association between the IRscore and disease state of patients with COVID-19, we examined the power of the IRscore to predict patient responses to immunotherapies. As shown in Fig. 7G, after 7 days of treatment with the IL-1 receptor blocker anakinra, every patient with COVID-19 presented a decrease in IRscore, suggesting that the established IRscore model might be useful for therapeutic efficacy prediction in COVID-19.

## Discussion

Immune homeostasis tuned by IRs is critical during viral infection^52^. The association between individual dysregulated IRs and the severity of COVID-19 has been mentioned in a few studies^15^. However, few studies have comprehensively identified immune modulation, immune-associated metabolic reprogramming, and clinical characterization of COVID-19 mediated by the integrated effects of multiple IRs. Furthermore, the mechanism by which IRs regulate the immune system to control infection remains controversial. In this study, we demonstrated that the integrated effects of multiple IRs defined immune and metabolic characteristics, which could be used to determine the clinical outcomes of patients with COVID-19. Our study also indicated that the integrated effect of IRs influenced the disease state of patients with COVID-19 through mechanisms other than inhibiting T cell activation. Identifying the correlation of IR patterns with immune cell infiltration and COVID-19 clinical features could contribute to enhancing our understanding of IRs in modulating immune responses and determining patient outcomes, as well as developing more effective therapeutic strategies for patients with COVID-19.

In this study, we identified two distinct IR patterns based on 42 IRs. The two patterns had significantly distinct disease severity, immune response characterization, and metabolic adaptation. Previous studies have proposed that lymphopenia and higher serum levels of CRP, D-dimers, ferritin, and lactate can be considered risk factors for severe COVID-19^53^. Consistent with this evidence, IR_cluster2 patients with higher serum levels of CRP, D-dimers, ferritin, and lactate and lower frequencies of CD8^+^ T cells required longer hospital stay, ICU, and mechanical ventilator support. Arunachalam et al. found that the myeloid cells of patients with severe COVID-19 exhibited reduced human leukocyte antigen class DR (HLA-DR) expression^21^. As expected, an analogous reduction in HLA-DRA, HLA-DRB1, HLA-DRB3, and HLA-DRB5 levels was observed in PBMCs with an IR pattern with poorer prognosis. Another feature of the patients with the most severe COVID-19 is their enhanced levels of proinflammatory cytokines in the plasma. Among these proinflammatory cytokines, only S100A12, the gene encoding EN-RAGE, is substantially enhanced in the PBMCs of patients with COVID-19 patients, while its gene expression in PBMCs has been confirmed to be consistent with the protein level in plasma. Furthermore, a previous study suggested that S100A12 is a biomarker of pulmonary damage involved in the pathogenesis of sepsis-induced ARDS^54–56^. Our discoveries pertaining to the significant upregulation of S100A12 in IR_cluster2 implied that IR_cluster2 patients might have developed pulmonary injury, resulting in poor survival. Therefore, IR_cluster2 patients presented with a suppressed immune state and were prone to pulmonary damage, which might explain their poor disease outcomes.

Using metabolic expression subtype classification analysis, we clarified that IR_cluster1 was characterized by upregulated energy production, amino acid metabolism, and nucleotide metabolism compared to IR_cluster2. In IR_cluster1, the increased levels of PDK1, LAT, LCK, and ZAP70 indicated the initiation of a faster energy production route (glycolysis) to activate T cells in the absence of increased glucose uptake^57,58^. Previous studies have suggested that activated CD8^+^ T cells rely on glycolysis to break down glucose to fuel different metabolic synthesis pathways. The upregulation of NFATC1 and NFATC2 implies the enhancement of glycolytic metabolism in patients in IR_cluster1^59,60^. The elevated expression of CD28 and AKT1 in IR_cluster1 also indicated the maintenance of enhanced glycolysis by PI3K-AKT signaling downstream of CD28 co-stimulation in IR_cluster1 patients^57,61,62^. To further satisfy nutrient demands, activated CD8^+^ T cells should increase the expression of solute carrier (SLC) transporters. We discovered the upregulation of SLC38A1, SLC3, and SLC7 subfamily members in IR_cluster1. SLC38A1, a glutamine transporter, is upregulated upon T cell activation in a CD28-dependent manner to supply carbons for the TCA cycle, which breaks down acetyl-CoA to carbon intermediates for producing energy and synthesizing new metabolic products^63^. SLC3 and SLC7 subfamily members regulate the uptake of large neutral amino acids to sustain protein synthesis in CD8^+^ T cells^64,65^. Therefore, the upregulation of these SLC transporters suggested the notion that IR_cluster1 patients satisfy the bioenergetic demands of CD8^+^ T cells for proliferation and differentiation by boosting glutamine uptake and its downstream metabolism.

Indeed, glutamine is another important nutrient that dictates the activation and function of immune cells. Glutaminolysis fueled by MYC is required to promote nucleotide synthesis to facilitate CD8^+^ T cell growth and proliferation^59^. We found that MYC was strengthened in IR_cluster1, indicating the initiation of glutaminolysis. Although MYC plays a crucial role in establishing metabolic reprogramming for T cell activation, another TCR-induced transcription factor, IRF4, is necessary for activated T cells to maintain their metabolic activity^66^. Similar to MYC, the expression of IRF4 was also remarkably ascending in IR_cluster1, indicating the sustained metabolic activity for T cell activation. To synchronize nutrient availability with demand, cells use the evolutionarily conserved nutrient-sensitive signaling complex mTOR to effectively control their growth, survival, and metabolism^67^. Congruent with its essential role in coordinating metabolic adaptation and immune cell fate, we noticed increased mTOR expression in IR_cluster1. In addition to glucose, amino acid, and nucleotide metabolism, CD8^+^ T cells also require fatty acid synthesis regulated by the activity of acetyl-CoA carboxylase alpha (ACACA) to support their proliferation and survival^68,69^. As expected, patients in IR_cluster1 displayed noticeable upregulation of ACACA. A previous study proposed that mitochondrial ROS production, T cell expansion, and effector function are suppressed by the lack of UQCRFS1 in vivo^70^. The high expression of UQCRFS1 in IR_cluster1 may contribute to facilitating mitochondrial ROS production, leading to the expansion and effector function of T cells, thus stimulating the effective immune response of IR_cluster1 patients to SARS-CoV-2 infection. In this study, we observed that IR_cluster1 upregulated the regulators of both effector T cell activation and metabolic pathways. The upregulation of these regulators contributed to enhanced nutrient metabolism for T cell activation and increased infiltration of CD8^+^ T cells, thus determining better prognostic outcomes of IR_cluster1 patients. Therefore, the immune response was intertwined with metabolic processes to ultimately determine the capability of the organism to provide an effective immune response and restore homeostasis.

In our study, the distinct disease severity, immune landscape, and metabolic characteristics between the two IR patterns were undoubtedly closely associated with the expression profile of 42 IRs. A striking increase in most IRs in IR_cluster1 with better prognosis was seemingly at odds with the literature describing an obvious upregulation of specific IRs in patients with severe COVID-19. However, in our scenario, patients in IR_cluster1 displayed not only an enhanced expression of IRs but also an increased frequency of immune effector cells (e.g., CD8^+^ T cells and activated CD4^+^ T memory cells) and an enhanced enrichment of antiviral immune pathways, such as the T and B cell receptor signaling pathway, NK cell-mediated cytotoxicity, antigen processing and presentation pathway, chemokine signaling pathway, cytokine–cytokine receptor interaction pathway, interferon signaling pathway, and NF-κB signaling pathway. Moreover, IR_cluster1 patients also showed significant inhibition of the biomarker of pulmonary damage, S100A12. Taken together, these results indicated that the physiological function of IRs during SARS-CoV-2 infection was not to simply dampen the innate or adaptive immune activation triggered by viral infection, but to maintain homeostasis to rapidly clear pathogens and effectively prevent immunopathology. Previous evidence proposes that the impact of IRs on cellular function is dependent on the strength of the inhibitory signal relative to the activation signal given within a certain time window^71^. Suppression of the immune response by IRs may have a disadvantageous effect on pathogen clearance, but without the activation-induced negative feedback mechanism regulated by IRs, the termination of effector mechanism activation could be delayed, thus increasing the chance of developing immunopathology. Therefore, additional regulation through IR-induced negative feedback ensures pathogen clearance with reduced effects owing to timely inhibition of the late activation signal. By analogy, the vigorous upregulation of most IRs in IR_cluster1 might reflect an immune activation-induced negative feedback mechanism to prevent rampant systemic immune hyperactivation. This negative feedback sustained more effective pathogen clearance, maintained organism homeostasis, and decreased the cost of the immune response, which guaranteed a favorable prognosis for patients in IR_cluster1. This also suggests that clinicians should consider whether patients with cancer who are also infected with SARS-CoV-2 are at increased risk of COVID-19-related immunopathology mediated by IR suppression while benefiting from immune checkpoint drug administration.

After determining the correlation between IR patterns and disease severity, immune status, and metabolic characteristics of patients with COVID-19, we further extracted the DEGs between distinct IR patterns, namely IR-related signature genes. Based on these genes, we identified two genomic subtypes correlated with IR patterns and COVID-19 patient prognosis, further confirming the existence of two IR-associated subtypes in COVID-19. Considering the heterogeneity of IR regulation in individuals, we constructed a scoring system to evaluate the IR pattern of individual patients and termed this system IRscore. The IR pattern with more severe disease presented a higher IRscore, whereas the pattern with better prognostic outcome exhibited a lower IRscore. Subsequently, we verified the reliability and stability of the IRscore model in predicting disease severity and staging in the testing cohort, LPS-induced inflammation model, and other datasets (GSE198256 and GSE152418).

Considering the close association between IRscore and disease severity, we further investigated the ability of IRscore to evaluate therapeutic effects. Immunotherapies have been used to treat patients with moderate COVID-19 who are admitted to general medicine wards^72^. Anakinra is considered for patients who do not require oxygen therapy but have high expression of inflammation-related biomarkers^73^. In the GSE163317 dataset, the IRscore decreased slightly after anakinra administration. We speculated that a more significant reduction in the IRscore might be observed if the treatment sample size were to be expanded. This also indicated that IRscore can be a reliable and powerful tool for precise treatment of patients with COVID-19. In summary, the IRscore showed predictive advantages in both COVID-19 disease status assessment and immunotherapy efficacy evaluation.

Although the importance of the integrated effect of 42 IRs in predicting the COVID-19 disease state has been elucidated, there remain some inadequacies in our study. One limitation was the requirement of further validation of our results in a prospective cohort to more scientifically define the cutoff value that could divide the high and low IRscores of patients with COVID-19. Additionally, because the IRscore model was established according to the transcriptomic levels of IRs in the PBMCs of patients with COVID-19, the model might not be able to use protein levels of other sample types to make accurate and stable predictions of disease state in patients with COVID-19. Therefore, confirmatory experiments of protein levels from other sample types are needed to determine the universality of this model and further validate our results.

## Conclusions

The present study demonstrated an extensive regulatory mechanism by which IRs affect the immune response, immune-associated metabolic adaption, and clinical features of COVID-19. The difference in IR patterns was a non-negligible factor leading to different clinical manifestations in individuals infected with SARS-CoV-2. The important role of the IR integrated effect in infection immunomodulation, disease status surveillance, and administration efficacy assessment was highlighted. As such, a theoretical basis that could aid future studies and facilitate the development of personalized and effective approaches for infectious diseases such as COVID-19 was presented.

### Supplementary Information

Supplementary Figures.

Supplementary Table S1.

Supplementary Table S2.

Supplementary Table S3.

Supplementary Table S4.

Supplementary Table S5.

## Data Availability

The datasets analyzed during the current study are all openly available in the GEO repository (https://www.ncbi.nlm.nih.gov/geo/) were GSE157103 (https://www.ncbi.nlm.nih.gov/geo/query/acc.cgi?acc=GSE157103), GSE198256 (https://www.ncbi.nlm.nih.gov/geo/query/acc.cgi?acc=GSE198256), GSE152418 (https://www.ncbi.nlm.nih.gov/geo/query/acc.cgi?acc=GSE152418), and GSE163317 (https://www.ncbi.nlm.nih.gov/geo/query/acc.cgi?acc=GSE163317).

## References

[1] WHO Coronavirus (COVID-19) dashboard, Mar. 2023 [online database]. https://covid19.who.int

[2] Berlin DA, Gulick RM, Martinez FJ (2020). Severe Covid-19. N. Engl. J. Med..

[3] Velavan TP, Pallerla SR, Ruter J, Augustin Y, Kremsner PG, Krishna S, Meyer CG (2021). Host genetic factors determining COVID-19 susceptibility and severity. EBioMedicine.

[4] Lowery SA, Sariol A, Perlman S (2021). Innate immune and inflammatory responses to SARS-CoV-2: Implications for COVID-19. Cell Host Microbe.

[5] Zheng M, Gao Y, Wang G, Song G, Liu S, Sun D, Xu Y, Tian Z (2020). Functional exhaustion of antiviral lymphocytes in COVID-19 patients. Cell. Mol. Immunol..

[6] Colonna M (1997). Immunoglobulin superfamily inhibitory receptors: From natural killer cells to antigen-presenting cells. Res. Immunol..

[7] Daeron M, Jaeger S, Du Pasquier L, Vivier E (2008). Immunoreceptor tyrosine-based inhibition motifs: A quest in the past and future. Immunol. Rev..

[8] Rumpret M, Drylewicz J, Ackermans LJE, Borghans JAM, Medzhitov R, Meyaard L (2020). Functional categories of immune inhibitory receptors. Nat. Rev. Immunol..

[9] Saresella M, Trabattoni D, Marventano I, Piancone F, La Rosa F, Caronni A, Lax A, Bianchi L, Banfi P, Navarro J (2021). NK cell subpopulations and receptor expression in recovering SARS-CoV-2 Infection. Mol. Neurobiol..

[10] Zheng HY, Zhang M, Yang CX, Zhang N, Wang XC, Yang XP, Dong XQ, Zheng YT (2020). Elevated exhaustion levels and reduced functional diversity of T cells in peripheral blood may predict severe progression in COVID-19 patients. Cell. Mol. Immunol..

[11] Kong Y, Wang Y, Wu X, Han J, Li G, Hua M, Han K, Zhang H, Li A, Zeng H (2020). Storm of soluble immune checkpoints associated with disease severity of COVID-19. Signal Transduct. Target. Ther..

[12] Shahbaz S, Xu L, Sligl W, Osman M, Bozorgmehr N, Mashhouri S, Redmond D, Perez Rosero E, Walker J, Elahi S (2021). The quality of SARS-CoV-2-specific T cell functions differs in patients with mild/moderate versus severe disease, and T cells expressing coinhibitory receptors are highly activated. J. Immunol..

[13] Herrmann M, Schulte S, Wildner NH, Wittner M, Brehm TT, Ramharter M, Woost R, Lohse AW, Jacobs T, Schulze Zur Wiesch J (2020). Analysis of co-inhibitory receptor expression in COVID-19 Infection compared to acute plasmodium falciparum Malaria: LAG-3 and TIM-3 correlate With T cell activation and course of disease. Front. Immunol..

[14] Yang J, Chang T, Tang L, Deng H, Chen D, Luo J, Wu H, Tang T, Zhang C, Li Z (2022). Increased expression of Tim-3 is associated with depletion of NKT cells in SARS-CoV-2 Infection. Front. Immunol..

[15] Saheb Sharif-Askari N, Saheb Sharif-Askari F, Mdkhana B, Al Heialy S, Alsafar HS, Hamoudi R, Hamid Q, Halwani R (2021). Enhanced expression of immune checkpoint receptors during SARS-CoV-2 viral infection. Mol. Ther. Methods Clin. Dev..

[16] Rha MS, Jeong HW, Ko JH, Choi SJ, Seo IH, Lee JS, Sa M, Kim AR, Joo EJ, Ahn JY (2021). PD-1-expressing SARS-CoV-2-specific CD8(+) T cells are not exhausted, but functional in patients with COVID-19. Immunity.

[17] Al-Mterin MA, Elkord E (2022). Inhibitory immune checkpoint receptors and ligands as prognostic biomarkers in COVID-19 patients. Front. Immunol..

[18] Crowley E, Di Nicolantonio F, Loupakis F, Bardelli A (2013). Liquid biopsy: Monitoring cancer-genetics in the blood. Nat. Rev. Clin. Oncol..

[19] Overmyer KA, Shishkova E, Miller IJ, Balnis J, Bernstein MN, Peters-Clarke TM, Meyer JG, Quan Q, Muehlbauer LK, Trujillo EA (2021). Large-scale multi-omic analysis of COVID-19 severity. Cell Syst..

[20] Brauns E, Azouz A, Grimaldi D, Xiao H, Thomas S, Nguyen M, Olislagers V, Vu Duc I, Orte Cano C, Del Marmol V (2022). Functional reprogramming of monocytes in patients with acute and convalescent severe COVID-19. JCI Insight.

[21] Arunachalam PS, Wimmers F, Mok CKP, Perera R, Scott M, Hagan T, Sigal N, Feng Y, Bristow L, Tak-Yin Tsang O (2020). Systems biological assessment of immunity to mild versus severe COVID-19 infection in humans. Science.

[22] Bertoni A, Penco F, Mollica H, Bocca P, Prigione I, Corcione A, Cangelosi D, Schena F, Del Zotto G, Amaro A (2022). Spontaneous NLRP3 inflammasome-driven IL-1-beta secretion is induced in severe COVID-19 patients and responds to anakinra treatment. J. Allergy Clin. Immunol..

[23] COvid-19 Multi-omics Blood ATlas (COMBAT) Consortium (2022). A blood atlas of COVID-19 defines hallmarks of disease severity and specificity. Cell.

[24] Kuhn M (2008). Building predictive models in R using the caret package. J. Stat. Softw..

[25] Wilkerson MD, Hayes DN (2010). ConsensusClusterPlus: A class discovery tool with confidence assessments and item tracking. Bioinformatics.

[26] Hanzelmann S, Castelo R, Guinney J (2013). GSVA: Gene set variation analysis for microarray and RNA-seq data. BMC Bioinform..

[27] Benjamini Y, Hochberg Y (1995). Controlling the false discovery rate—A practical and powerful approach to multiple testing. J. R. Stat. Soc. Ser. B Stat. Methodol..

[28] Yu G, Wang LG, Han Y, He QY (2012). clusterProfiler: An R package for comparing biological themes among gene clusters. OMICS.

[29] Newman AM, Liu CL, Green MR, Gentles AJ, Feng W, Xu Y, Hoang CD, Diehn M, Alizadeh AA (2015). Robust enumeration of cell subsets from tissue expression profiles. Nat. Methods.

[30] Peng X, Chen Z, Farshidfar F, Xu X, Lorenzi PL, Wang Y, Cheng F, Tan L, Mojumdar K, Du D (2018). Molecular characterization and clinical relevance of metabolic expression subtypes in human cancers. Cell Rep..

[31] Fabregat A, Sidiropoulos K, Garapati P, Gillespie M, Hausmann K, Haw R, Jassal B, Jupe S, Korninger F, McKay S (2016). The Reactome pathway Knowledgebase. Nucleic Acids Res..

[32] Anders S, Huber W (2010). Differential expression analysis for sequence count data. Genome Biol..

[33] Kursa MB, Rudnicki WR (2010). Feature selection with the boruta package. J. Stat. Softw..

[34] Sotiriou C, Wirapati P, Loi S, Harris A, Fox S, Smeds J, Nordgren H, Farmer P, Praz V, Haibe-Kains B (2006). Gene expression profiling in breast cancer: Understanding the molecular basis of histologic grade to improve prognosis. J. Natl. Cancer Inst..

[35] Xian H, Liu Y, Rundberg Nilsson A, Gatchalian R, Crother TR, Tourtellotte WG, Zhang Y, Aleman-Muench GR, Lewis G, Chen W (2021). Metformin inhibition of mitochondrial ATP and DNA synthesis abrogates NLRP3 inflammasome activation and pulmonary inflammation. Immunity.

[36] Feldman AT, Wolfe D (2014). Tissue processing and hematoxylin and eosin staining. Methods Mol. Biol..

[37] Zhang B, Wu Q, Li B, Wang D, Wang L, Zhou YL (2020). m(6)A regulator-mediated methylation modification patterns and tumor microenvironment infiltration characterization in gastric cancer. Mol. Cancer.

[38] Wang S, Xiong Y, Zhang Q, Su D, Yu C, Cao Y, Pan Y, Lu Q, Zuo Y, Yang L (2021). Clinical significance and immunogenomic landscape analyses of the immune cell signature based prognostic model for patients with breast cancer. Brief. Bioinform..

[39] Harrell FE, Lee KL, Mark DB (1996). Multivariable prognostic models: Issues in developing models, evaluating assumptions and adequacy, and measuring and reducing errors. Stat. Med..

[40] Zeng D, Li M, Zhou R, Zhang J, Sun H, Shi M, Bin J, Liao Y, Rao J, Liao W (2019). Tumor microenvironment characterization in gastric cancer identifies prognostic and immunotherapeutically relevant gene signatures. Cancer Immunol. Res..

[41] Heagerty PJ, Lumley T, Pepe MS (2000). Time-dependent ROC curves for censored survival data and a diagnostic marker. Biometrics.

[42] Sette A, Crotty S (2021). Adaptive immunity to SARS-CoV-2 and COVID-19. Cell.

[43] Huang C, Wang Y, Li X, Ren L, Zhao J, Hu Y, Zhang L, Fan G, Xu J, Gu X (2020). Clinical features of patients infected with 2019 novel coronavirus in Wuhan, China. Lancet.

[44] Reina-Campos M, Scharping NE, Goldrath AW (2021). CD8(+) T cell metabolism in infection and cancer. Nat. Rev. Immunol..

[45] Ganeshan K, Chawla A (2014). Metabolic regulation of immune responses. Annu. Rev. Immunol..

[46] Man K, Kallies A (2015). Synchronizing transcriptional control of T cell metabolism and function. Nat. Rev. Immunol..

[47] O'Brien KL, Finlay DK (2019). Immunometabolism and natural killer cell responses. Nat. Rev. Immunol..

[48] Lee EY, Kim S, Kim MH (2018). Aminoacyl-tRNA synthetases, therapeutic targets for infectious diseases. Biochem. Pharmacol..

[49] Lee EY, Lee HC, Kim HK, Jang SY, Park SJ, Kim YH, Kim JH, Hwang J, Kim JH, Kim TH (2016). Infection-specific phosphorylation of glutamyl-prolyl tRNA synthetase induces antiviral immunity. Nat. Immunol..

[50] Liang D, Tian L, You R, Halpert MM, Konduri V, Baig YC, Paust S, Kim D, Kim S, Jia F (2017). AIMp1 potentiates TH1 polarization and is critical for effective antitumor and antiviral immunity. Front. Immunol..

[51] Ahn YH, Park S, Choi JJ, Park BK, Rhee KH, Kang E, Ahn S, Lee CH, Lee JS, Inn KS (2016). Secreted tryptophanyl-tRNA synthetase as a primary defence system against infection. Nat. Microbiol..

[52] Blackburn SD, Shin H, Haining WN, Zou T, Workman CJ, Polley A, Betts MR, Freeman GJ, Vignali DA, Wherry EJ (2009). Coregulation of CD8+ T cell exhaustion by multiple inhibitory receptors during chronic viral infection. Nat. Immunol..

[53] Velavan TP, Meyer CG (2020). Mild versus severe COVID-19: Laboratory markers. Int. J. Infect. Dis..

[54] Zhang Z, Han N, Shen Y (2020). S100A12 promotes inflammation and cell apoptosis in sepsis-induced ARDS via activation of NLRP3 inflammasome signaling. Mol. Immunol..

[55] Zhao F, Hoechst B, Duffy A, Gamrekelashvili J, Fioravanti S, Manns MP, Greten TF, Korangy F (2012). S100A9 a new marker for monocytic human myeloid-derived suppressor cells. Immunology.

[56] Pena OM, Hancock DG, Lyle NH, Linder A, Russell JA, Xia J, Fjell CD, Boyd JH, Hancock RE (2014). An endotoxin tolerance signature predicts sepsis and organ dysfunction at initial clinical presentation. EBioMedicine.

[57] Menk AV, Scharping NE, Moreci RS, Zeng X, Guy C, Salvatore S, Bae H, Xie J, Young HA, Wendell SG (2018). Early TCR signaling induces rapid aerobic glycolysis enabling distinct acute t cell effector functions. Cell Rep..

[58] Cammann C, Rath A, Reichl U, Lingel H, Brunner-Weinzierl M, Simeoni L, Schraven B, Lindquist JA (2016). Early changes in the metabolic profile of activated CD8(+) T cells. BMC Cell Biol..

[59] Wang R, Dillon CP, Shi LZ, Milasta S, Carter R, Finkelstein D, McCormick LL, Fitzgerald P, Chi H, Munger J (2011). The transcription factor Myc controls metabolic reprogramming upon T lymphocyte activation. Immunity.

[60] Klein-Hessling S, Muhammad K, Klein M, Pusch T, Rudolf R, Floter J, Qureischi M, Beilhack A, Vaeth M, Kummerow C (2017). NFATc1 controls the cytotoxicity of CD8(+) T cells. Nat. Commun..

[61] Frauwirth KA, Riley JL, Harris MH, Parry RV, Rathmell JC, Plas DR, Elstrom RL, June CH, Thompson CB (2002). The CD28 signaling pathway regulates glucose metabolism. Immunity.

[62] Jacobs SR, Herman CE, Maciver NJ, Wofford JA, Wieman HL, Hammen JJ, Rathmell JC (2008). Glucose uptake is limiting in T cell activation and requires CD28-mediated Akt-dependent and independent pathways. J. Immunol..

[63] Carr EL, Kelman A, Wu GS, Gopaul R, Senkevitch E, Aghvanyan A, Turay AM, Frauwirth KA (2010). Glutamine uptake and metabolism are coordinately regulated by ERK/MAPK during T lymphocyte activation. J. Immunol..

[64] Fotiadis D, Kanai Y, Palacin M (2013). The SLC3 and SLC7 families of amino acid transporters. Mol. Aspects Med..

[65] Sinclair LV, Rolf J, Emslie E, Shi YB, Taylor PM, Cantrell DA (2013). Control of amino-acid transport by antigen receptors coordinates the metabolic reprogramming essential for T cell differentiation. Nat. Immunol..

[66] Man K, Miasari M, Shi W, Xin A, Henstridge DC, Preston S, Pellegrini M, Belz GT, Smyth GK, Febbraio MA (2013). The transcription factor IRF4 is essential for TCR affinity-mediated metabolic programming and clonal expansion of T cells. Nat. Immunol..

[67] Powell JD, Delgoffe GM (2010). The mammalian target of rapamycin: Linking T cell differentiation, function, and metabolism. Immunity.

[68] Lee J, Walsh MC, Hoehn KL, James DE, Wherry EJ, Choi Y (2014). Regulator of fatty acid metabolism, acetyl coenzyme a carboxylase 1, controls T cell immunity. J. Immunol..

[69] Ibitokou SA, Dillon BE, Sinha M, Szczesny B, Delgadillo A, Reda Abdelrahman D, Szabo C, Abu-Elheiga L, Porter C, Tuvdendorj D (2018). Early inhibition of fatty acid synthesis reduces generation of memory precursor effector T cells in chronic infection. J. Immunol..

[70] Sena LA, Li S, Jairaman A, Prakriya M, Ezponda T, Hildeman DA, Wang CR, Schumacker PT, Licht JD, Perlman H (2013). Mitochondria are required for antigen-specific T cell activation through reactive oxygen species signaling. Immunity.

[71] Ravetch JV, Lanier LL (2000). Immune inhibitory receptors. Science.

[72] van de Veerdonk FL, Giamarellos-Bourboulis E, Pickkers P, Derde L, Leavis H, van Crevel R, Engel JJ, Wiersinga WJ, Vlaar APJ, Shankar-Hari M (2022). A guide to immunotherapy for COVID-19. Nat. Med..

[73] Kyriazopoulou E, Poulakou G, Milionis H, Metallidis S, Adamis G, Tsiakos K, Fragkou A, Rapti A, Damoulari C, Fantoni M (2021). Early treatment of COVID-19 with anakinra guided by soluble urokinase plasminogen receptor plasma levels: A double-blind, randomized controlled phase 3 trial. Nat. Med..

