# Supplementary material for: Immune inhibitory receptor-mediated immune response, metabolic adaptation, and clinical characterization in patients with COVID-19
Source: Sci Rep. 2023 Nov 6;13:19221. doi: 10.1038/s41598-023-45883-w (PMC10628246; doi:10.1038/s41598-023-45883-w)
Supplement: Supplementary file 1 — Supplementary Figures. [file 41598_2023_45883_MOESM1_ESM.docx]

**
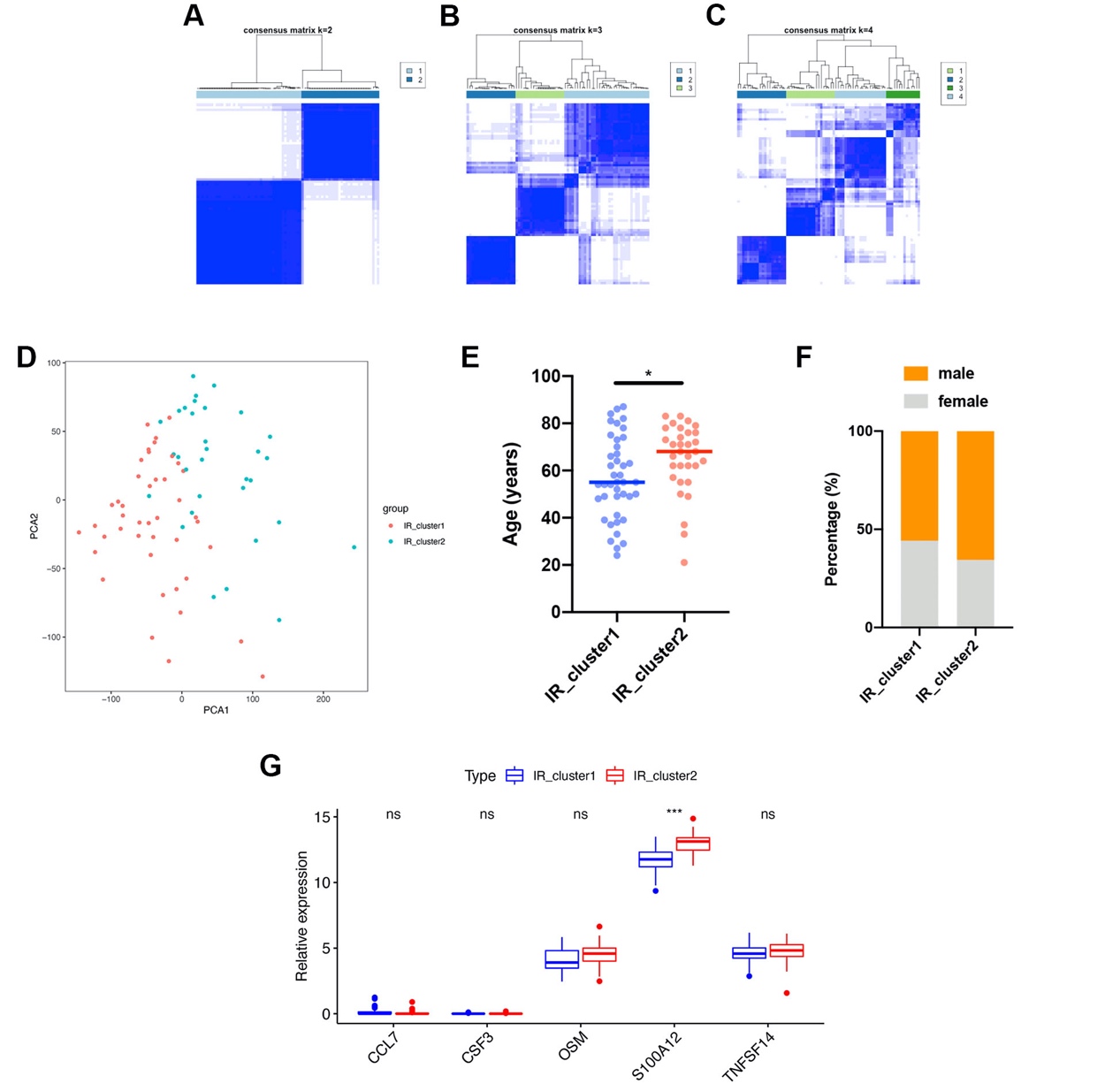
FIGURE S1**

**(A-C)** Unsupervised clustering of 42 IR genes and consensus matrices for k = 2–4. **(D)** Principal component analysis for the transcriptome profiles of two IR patterns, showing a remarkable difference on transcriptome profiles between two distinct IR clusters. **(E)** The differences in age between the two IR patterns in training cohort (Mann–Whitney U test). **(F)** The differences in sex proportions between the two IR clusters in training cohort (chi-square test). **(G)** Expression levels of proinflammatory cytokine genes between two IR patterns in training cohort (Mann–Whitney U test). The upper and lower ends of the boxes represent interquartile range of values. The lines in the boxes represent median value, and dots show outliers. The asterisks represent the statistical *P* value (**P* < 0.05; ***P* < 0.01; ****P* < 0.001; ns, no significance).


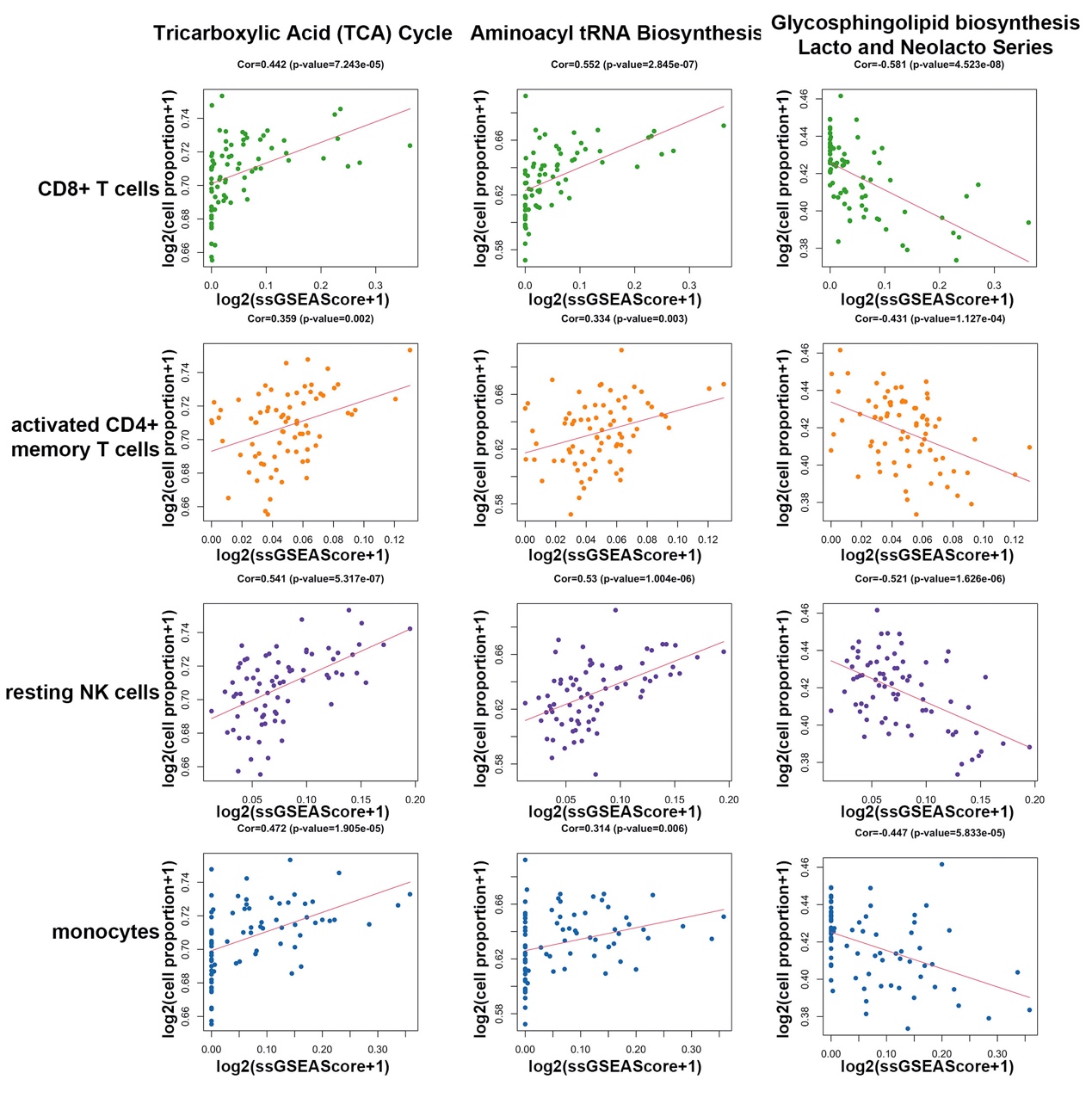


**FIGURE S2**

Correlation analysis between immune cell infiltration and cellular metabolic pathway enrichment. The correlation coefficient and *P* value of Spearman correlation analysis were presented at the top of each graph.

**
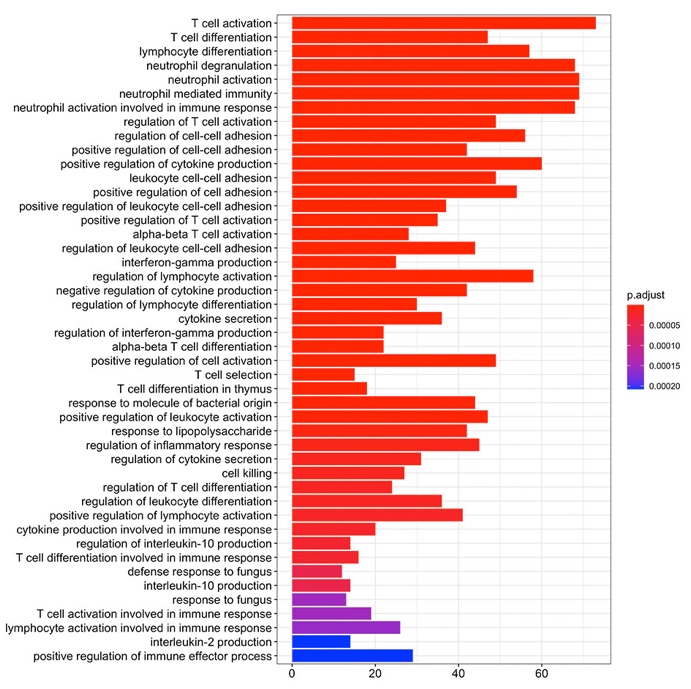
**

**FIGURE S3**

Functional annotation for IR phenotype associated genes through GO enrichment analysis. The x axis indicates the number of genes enriched within each GO term. The color depth of the bars represents the adjust *P* value of each GO term enriched.

**
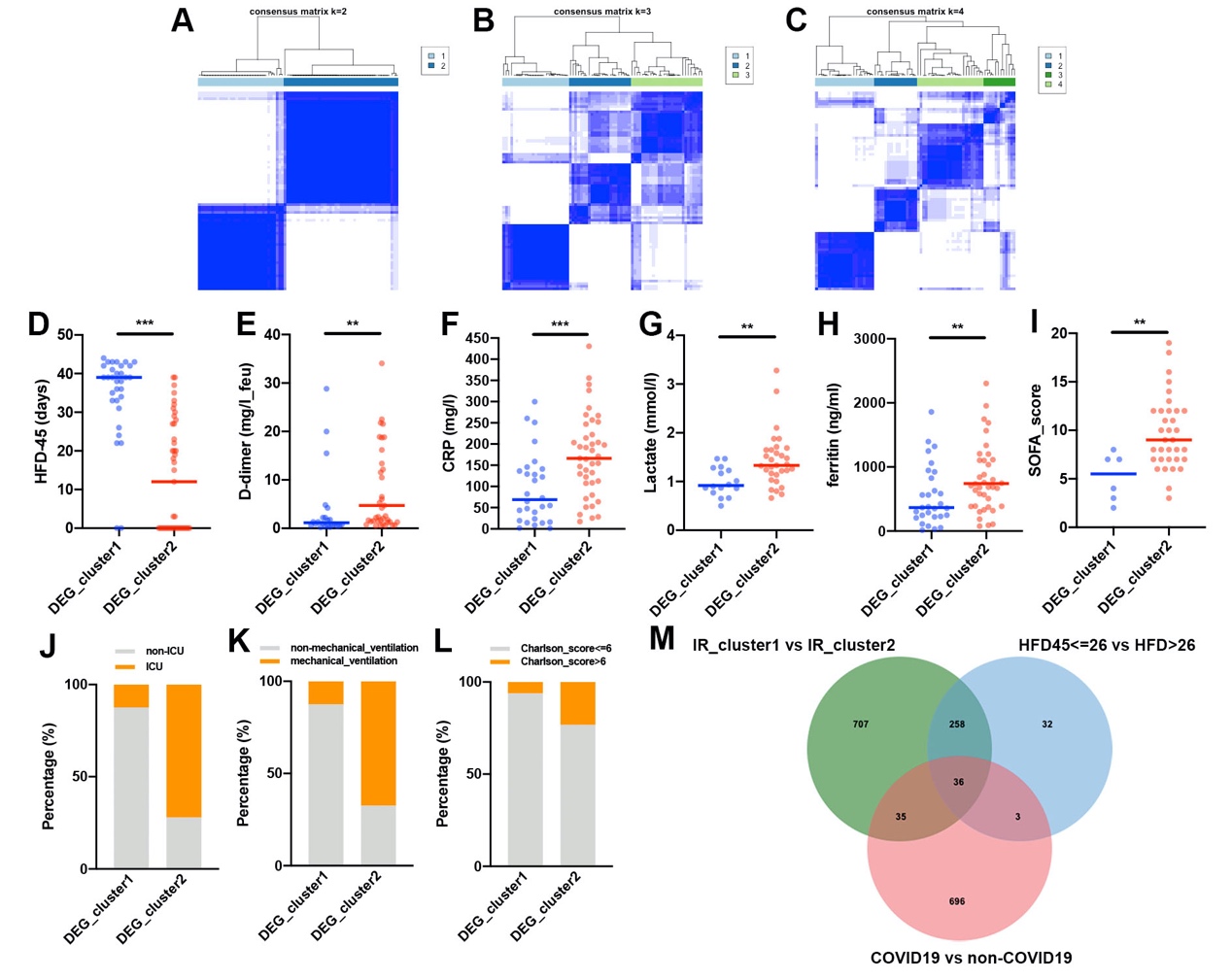
FIGURE S4**

**(A-C)** Unsupervised clustering of 1036 IR phenotype-related genes in the training cohort and consensus matrices for k = 2–4. **(D)** Differences in HFD-45 between the two DEG clusters in the training cohort (Mann–Whitney U test). **(E-H)** Differences in the concentrations of D-dimer (E), CRP (F), lactate (G), and ferritin (H) between the two DEG clusters in the training cohort (Mann–Whitney U test). (**I)** Differences in the SOFA scores between the two DEG clusters in training cohort (unpaired *t* test). **(J-L)** Differences in the proportions of ICU (J), mechanical ventilation (K), and Charlson score > 6 (L) between the two DEG clusters in training cohort (chi-square test). **(M)** Overlap analysis of the DE genes extracted from comparison between IR_cluster1 and IR_cluster2, COVID-19 and non-COVID-19 patients, HFD45 $\leq$ 26 and HFD45 >26 was shown in venn diagram. The asterisks represent the statistical *P* value (**P* < 0.05; ***P* < 0.01; ****P* < 0.001; ns, no significance).

**
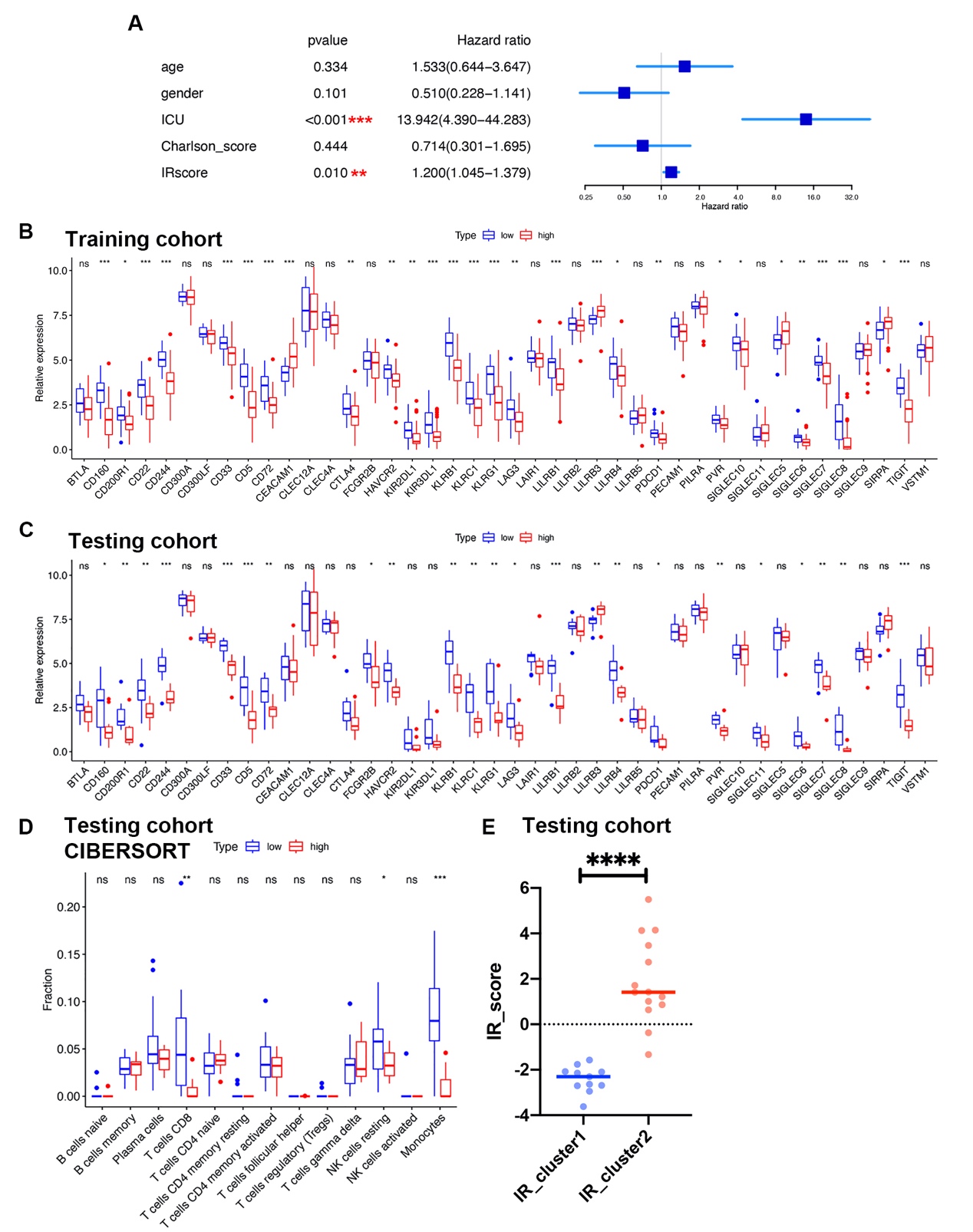
FIGURE S5**

**(A)** Forest plot displaying the multivariate Cox regression analysis results of IRscore in training cohort. **(B)** Expression levels of 42 IR genes between patients with the high and low IRscore in the training cohort (Mann–Whitney U test). The upper and lower ends of the boxes represent interquartile range of values. The lines in the boxes represent median value, and dots show outliers. **(C)** Expression levels of 42 IR genes between patients with the high and low IRscore in testing cohort (Mann–Whitney U test). The upper and lower ends of the boxes represent interquartile range of values. The lines in the boxes represent median value, and dots show outliers. **(D)** The abundance of immune cells calculated by CIBERSORT between distinct IR patterns in the testing cohort (Mann–Whitney U test). **(E)** Differences in the IRscore between the IR patterns in the testing cohort (Mann–Whitney U test). The asterisks represent the statistical *P* value (**P* < 0.05; ***P* < 0.01; ****P* < 0.001; ns, no significance).
